# Efficacy of stem cell therapy in patients with chronic liver disease: an umbrella review of systematic reviews

**DOI:** 10.1097/JS9.0000000000001644

**Published:** 2024-05-22

**Authors:** Yue Teng, Abhay M. Gaidhane, Bijaya K. Padhi, Quazi S. Zahiruddin, Saad Alhumaid, Rakesh K. Sharma, Sarvesh Rustagi, Prakasini Satapathy, Divya Sharma, Mithhil Arora, Ali Hazazi, Amani Alturaifi, Mansoor A. AlRshoud, Ali A. Zaidan, Fadel A.M. Almosa, Suha A. Alzayer, Razi Al Alqam, Raghad Alhajaji, Ali A. Rabaan

**Affiliations:** a Faculty of Medicine, Health and Life Science, Swansea University, Swansea, UK; b Jawaharlal Nehru Medical College, and Global Health Academy, School of Epidemiology and Public Health, Datta Meghe Institute of Higher Education, Wardha, India; c Department of Community Medicine and School of Public Health, Postgraduate Institute of Medical Education and Research, Chandigarh, India; d South Asia Infant Feeding Research Network (SAIFRN), Division of Evidence Synthesis, Global Consortium of Public Health and Research, Datta Meghe Institute of Higher Education, Wardha, India; e School of Pharmacy, University of Tasmania, Hobart, Australia; f Graphic Era (Deemed to be University) Clement Town, Dehradun, India; g Graphic Era Hill University, Clement Town Dehradun, India; h School of Applied and Life Sciences, Uttaranchal University, Dehradun, Uttarakhand; i Center for Global Health Research, Saveetha Medical College and Hospital, Saveetha Institute of Medical and Technical Sciences, Saveetha University, Chennai; j Medical Laboratories Techniques Department, Al-Mustaqbal University, Hillah, Babil, Iraq; k Centre of Research Impact and Outcome, Chitkara University, Rajpura Punjab, India; l Chitkara Centre for Research and Development, Chitkara University, Himachal Pradesh, India; m Department of Pathology and Laboratory Medicine, Security Forces Hospital Program; n College of Medicine, Alfaisal University; o Laboratory Total Quality Management Department, Riyadh Regional Laboratory, Riyadh, Saudi Arabia; p Gastroenterology Department, King Fahad Armed Forces Hospital, Jeddah, Saudi Arabia; q Gastroenterology Unit, Department of Internal Medicine, Qatif Central Hospital, Ministry of Health, Qatif, Saudi Arabia; r Laboratory Department, Qatif Comprehensive Inspection Center, Qatif, Saudi Arabia; s Department Minister of Enterprise, University Hospital Limerick, Limerick V94 Kty0,Ireland; t Family Medicine Section, Primary Healthcare Department, Makkah Health Cluster, Ministry of Health, Makkah, Saudi Arabia; u Molecular Diagnostic Laboratory, Johns Hopkins Aramco Healthcare, Dhahran, Saudi Arabia; v Department of Public Health and Nutrition, The University of Haripur, Haripur, Pakistan

**Keywords:** chronic liver disease, liver cirrhosis, meta-analysis, stem cells, umbrella review

## Abstract

**Background:**

Stem cell therapy offers promising benefits like modulating immune responses, reducing inflammation, and aiding liver regeneration. This umbrella review seeks to compile evidence from systematic reviews to assess the efficacy of stem cell therapy for improving liver function and survival rates in chronic liver disease patients.

**Methods:**

We searched electronic databases up to February 15, 2024. The selection process focused on systematic reviews comparing stem cell therapy with standard care or a placebo. The primary outcomes evaluated were changes in liver enzymes, the Model for End-Stage Liver Disease score, and survival rates. Nested Knowledge software was utilized for screening and data extraction. All statistical analyses were performed using R software, version 4.3.

**Results:**

Our umbrella review included 28 systematic reviews. The meta-analysis showcased a notable improvement in survival rates with a pooled relative risk of 1.487 [95% confidence interval (CI): 1.281–1.727). In nonrandomized studies, albumin levels exhibited a standardized mean difference (SMD) of 0.786 (95% CI: 0.368–1.204), indicating positive therapeutic effects. For alanine aminotransferase, the meta-analysis revealed a decrease in levels with an SMD of −0.499 (95% CI: −0.834 to −0.164), and for aspartate aminotransferase, an overall SMD of −0.362 (95% CI: −0.659 to −0.066) was observed, suggesting hepatoprotective effects. No significant changes were observed in total bilirubin levels and Model for End-Stage Liver Disease scores in randomized controlled trials.

**Conclusion:**

Stem cell therapy exhibits potential as a novel treatment for chronic liver diseases, as it has demonstrated improvements in survival rates and certain liver function markers. More high-quality randomized controlled trials are needed in the future to fully ascertain the efficacy of stem cell therapy in this patient population.

## Introduction

Highlights

- Stem cell therapy shows a significant improvement in survival rates for chronic liver disease patients, with a 48.7% increase compared to standard treatments.
- There were no notable changes in the Model for End-Stage Liver Disease (MELD) scores or total bilirubin levels in randomized controlled trials (RCTs), suggesting selective benefits of stem cell therapy.
- Decreased levels of liver enzymes alanine aminotransferase (ALT) and aspartate aminotransferase (AST) in patients indicate potential hepatoprotective effects of stem cell treatments.
- High study heterogeneity and publication bias in non-RCTs emphasize the need for more standardized, high-quality clinical trials.

Liver cirrhosis, characterized as the final stage of liver scarring due to various liver conditions such as hepatitis and chronic alcohol abuse, poses a significant global health concern^1–3^. The pathophysiology of liver cirrhosis involves a complex interplay of cellular and molecular mechanisms leading to liver injury, inflammation, fibrosis, and, eventually, liver failure^4^. Traditional treatments for liver cirrhosis, including lifestyle modifications, medication, and liver transplantation, offer restricted efficacy and come with various drawbacks^5–7^. Liver transplantation, while effective, is severely limited by the availability of donor organs, surgical risks, and the need for lifelong immunosuppression^8,9^. Consequently, there has been a substantial interest in alternative therapeutic strategies that can halt the progression of liver disease or even reverse liver damage.

Stem cell therapy has emerged as a promising approach in regenerative medicine, offering potential benefits for various diseases, including liver cirrhosis^10,11^. Mesenchymal stem cells (MSCs) are multipotent stromal cells that can evolve into a range of cell types, including hepatocytes. These cells are obtainable from multiple origins, including adipose tissue, bone marrow, blood, and umbilical cord, making them readily accessible for therapeutic purposes^12^. The therapeutic potential of MSCs in liver cirrhosis is attributed to their ability to modulate immune responses, reduce inflammation, and promote the regeneration of liver tissue with various mechanisms, including the secretion of paracrine factors that enhance tissue repair and reduce fibrosis^13,14^.

Despite the promising potential of stem cell therapy, its clinical application in liver cirrhosis has been met with both enthusiasm and caution. The enthusiasm stems from the growing body of preclinical and early clinical evidence suggesting that stem cell therapy may improve liver function, reduce fibrosis, and enhance the quality of life for patients with liver diseases^15,16^. However, caution is warranted due to the variability in study designs, stem cell sources, and delivery methods, as well as the need for a deeper understanding of such treatments’ long-term safety and efficacy^17^. Recent systematic reviews have sought to consolidate the evidence regarding the efficacy of stem cell therapy for chronic or end-stage liver diseases such as liver cirrhosis, offering a more comprehensive understanding of its therapeutic potential and limitations^18–25^. These reviews typically focus on RCTs to assess the impact of stem cell therapy on liver function, as measured by standard liver function tests and scores such as the MELD score, albumin levels, and markers of liver inflammation and fibrosis^18–25^.

The rationale behind this umbrella review stems from the fragmented evidence regarding the efficacy of stem cell therapy in chronic liver diseases. While individual studies and systematic reviews offer valuable insights, there still needs to be a gap in synthesizing this information across a broader spectrum of research. This review aims to provide a bird’s eye view of this topic by conducting an overarching analysis of systematic reviews and meta-analyses, thereby providing a comprehensive evaluation of stem cell therapy’s current state in treating liver cirrhosis. The objective is to assess the efficacy of stem cell therapy in improving liver function and reducing fibrosis in patients with liver cirrhosis and other chronic liver diseases. Through this review, we aim to offer a clearer understanding of the potential role of stem cell therapy in liver cirrhosis, guiding future research directions and clinical applications.

## Methods

This umbrella review adhered to the Joanna Briggs Institute methodology for conducting umbrella reviews^26^ and complied with the Reporting Items for Systematic Reviews and Meta-Analyses (PRISMA) standards (Table S1, Supplemental Digital Content 1, http://links.lww.com/JS9/C640)^27^. The review protocol was registered with PROSPERO. The Nested Knowledge web application (Nested Knowledge, MN, USA) was utilized for both the screening of studies and the extraction of data.

### Eligibility criteria

In our umbrella review, we aimed to evaluate the efficacy of stem cell therapy in patients with liver cirrhosis and other chronic liver diseases. To be included in our review, studies needed either RCTs or nonrandomized human studies that compared stem cell therapy with standard care or a placebo. We did not impose any restrictions on the type of stem cells used, their dosage, the frequency of administration, or the method of administration. Our primary outcomes of interest were changes in liver enzymes, the MELD score, and survival rate. We excluded single primary studies, nonsystematic reviews, commentary articles, and studies not published in peer-reviewed journals from our analysis. Additionally, studies focused on nonhuman subjects were also excluded from consideration.

### Information sources and search strategy

To identify pertinent systematic reviews and meta-analyses, searches spanned several electronic databases, such as PubMed, EMBASE, Cochrane Library, and Web of Science, covering the period from their inception up to February 15, 2024. The search strategy was crafted with the help of a librarian who is experienced in conducting systematic reviews. It encompassed a mix of Medical Subject Headings terms and free-text terms related to “stem cell therapy” and “liver cirrhosis,” combined with “systematic review OR meta-analysis.” Language, article type, and publication date were not restricted through filters. Details of the search strategy can be found in Table S2, Supplemental Digital Content 1, http://links.lww.com/JS9/C640.

### Selection process

Two independent evaluators (P.S., B.K.P.) conducted a screening of titles and abstracts to determine their suitability. Subsequently, the complete texts of those reviews deemed potentially eligible were obtained and thoroughly assessed against the inclusion criteria. In instances of disagreement between the initial reviewers, resolutions were sought through discussions or by involving a third reviewer for an objective decision. The Nested Knowledge software was utilized to streamline and assist in the screening procedure.

### Data collection and quality assessment

A standardized data extraction form was used to collect data from the included systematic reviews. Extracted information included author name, year of publication, database, date of searches, types of included studies, outcomes, risk of bias, key results, types of stem cell therapy, comparison interventions, outcomes assessed, and main findings. The Nested Knowledge software was utilized for data extraction by using the “tagging” function.

The quality of the included systematic reviews and meta-analyses was evaluated using the validated tool AMSTAR 2^28^. This tool evaluated several aspects of the review methodology, including the comprehensiveness of the literature search, the risk of bias in included studies, and the appropriateness of meta-analytical methods.

### Data analysis

Data were synthesized narratively to underline stem cell therapy’s efficacy outcomes in liver diseases, as reported across the reviews. Meta-analysis was considered and conducted for outcomes where the data across reviews were found to be sufficiently homogeneous. Each specified outcome was analyzed separately by aggregating the findings from RCTs or nonrandomized studies using R statistical software, version 4.3^29–31^. In the case of binary outcomes, the analysis combined the event counts in the stem cell and comparison groups with the total number of participants to calculate the relative risk (RR) and the corresponding confidence intervals (CIs)^32–35^. Mean values and SDs were utilized for continuous outcomes. The degree of heterogeneity among the study results was determined using the *I* ^2^ and Tau^2^ statistics, and a prediction interval was also estimated. Funnel plots served as the method for evaluating potential publication bias, with a statistical significance threshold set at a *P* value below 0.05.

## Results

### Literature search

**Figure 1:**
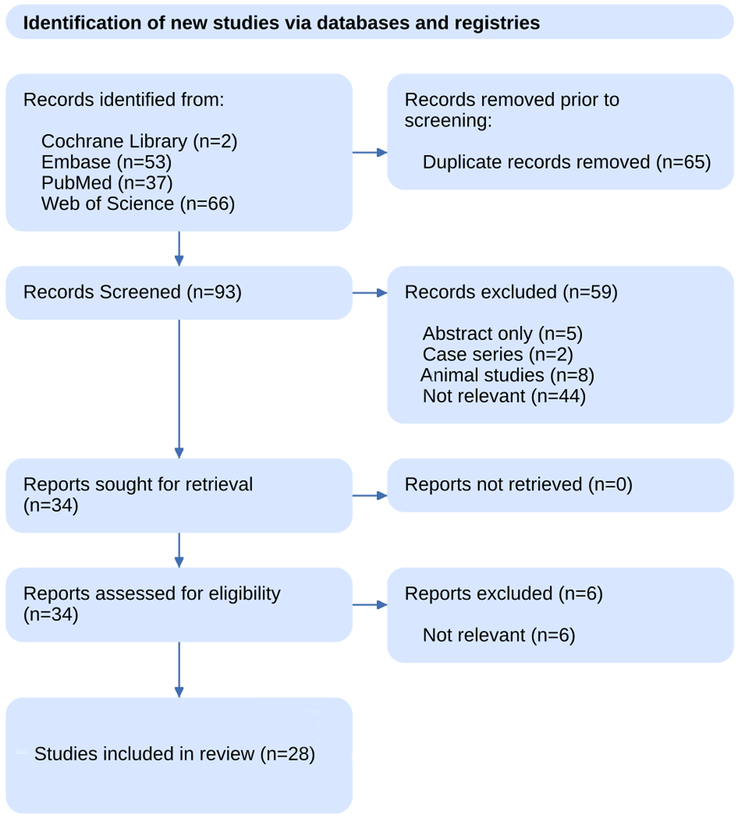
PRISMA flowchart depicting study selection process.

The literature search resulted in 158 records being identified from various databases. Before screening, 65 duplicate records were removed, leaving 93 records to be screened. During the screening phase, 59 records were excluded: five were abstracts only, two were case series, eight were animal studies, and 44 were deemed irrelevant. Of the remaining records, 34 reports were sought for retrieval, and all 34 reports were successfully retrieved and assessed for eligibility. At this stage, six reports were excluded as irrelevant, resulting in 28 studies^18–25,30,36–54^ included in the systematic review for the umbrella review (Fig. 1).

### Summary of included systematic reviews

The summary characteristics of the included studies are displayed in Table 1. Twenty-eight systematic reviews were included^18–25,30,36–54^, evaluating the efficacy of various stem cell therapies and interventions such as granulocyte colony-stimulating factor for treating liver diseases, specifically liver cirrhosis, acute-on-chronic liver failure, and end-stage liver disease. The studies were retrieved from several databases, including PubMed, MEDLINE, Cochrane Library, EMBASE, Web of Science, and others, with the search timelines extending up to the year 2023 for some reviews. The interventions reviewed encompassed therapies with bone marrow-derived MSCs, autologous stem cell transplantation, and other stem cell types, including umbilical cord-derived MSCs. Outcomes assessed across these reviews included liver function tests such as MELD score, albumin, bilirubin, prothrombin time, as well as survival rates and quality of life. The number of included studies within these reviews varied, with some analyzing as few as two and others up to 39 individual studies. Study types ranged from RCTs to observational studies, case–control studies, and cohort studies. Types of diseases targeted were mostly liver cirrhosis and acute-on-chronic liver failure, with various underlying causes such as viral hepatitis. Most reviews pointed to the therapeutic benefit of stem cell therapies in improving liver function, with some highlighting improvements in short-term survival rates. Several reviews indicated that the therapies were generally safe and could be effective as standalone treatments or adjuncts to conventional therapy. However, the risk of bias within included studies varied, with some systematic reviews reporting high-quality studies and others indicating moderate to high risks of bias. The quality of the reviews is given in Table S3, Supplemental Digital Content 1, http://links.lww.com/JS9/C640.

**Table 1:** Summary of characteristics of included studies.

References	Databases and search	Number of included studies	Type of studies	Type of disease	Type of intervention	Outcomes	Risk of bias of included studies	Key findings	Publication bias
AdiwinataPawitan^37^	PubMed/MEDLINE and Cochrane Library Up to16 June 2018	25	RCTs and nonrandomized control trials	Liver failure	Various adult stem cells	ALB, AST, TBIL, PT	NA	BM-MSCs outperformed other adult stem cells, but no study demonstrated complete and sustainable outcomes. IV administration was as effective as invasive routes.	NA
Chavez-Tapia *et al*.^19^	CENTRAL, MEDLINE, EMBASE and LILAC Up to November 2013	2	RCTs	ACLF	G-CSF	MELD score, mortality, adverse events	Low risk	The use of G-CSF in treating patients with ACLF significantly decreased short-term mortality. However, G-CSF did not reduce mortality associated with gastrointestinal bleeding	NA
Chen^40^	PubMed and EMBASE	3	RCTs	hepatitis B virus-ACLF	Mesenchymal stem cells	TBIL, Mortality, MELD score	NA	MSC treatment significantly reduces mortality rates without increasing the incidence of severe complications	NA
Chen^39^	Medline, Embase, SinoMed, and Cochrane Library Up to 5 August 2018	8	RCTs, cohort studies	Viral hepatitis-induced liver cirrhosis	Autologous stem cell transplantation	ALB, AST, PT, MELD score, CPS, Adverse events	Moderate	Autologous stem cell transplantation may benefit patients with viral hepatitis-induced LC and is considered relatively safe for them	Not suspected
Huang *et al*.^24^	Cochrane Library, PubMed, EMBASE, and China Biology Medicine disc up to January 2020	6	Nonrandomized controlled study	ACLF	G-CSF	MELD, ALT, ALB, AST, TBIL, PTA/PT, INR, survival rates	Low risk	G-CSF could be beneficial and effective in treating ACLF	No publication bias
Kim *et al*.^25^	Ovid-MEDLINE, EMBASE, and Cochrane Library Up to November 2014	14	Controlled trials and before-after studies	Chronic liver diseases	Mesenchymal stem cells	ALB, TBIL, MELD	High risk of bias	MSC transplantation is deemed safe for treating chronic liver disease	Not suspected
Konstantis^36^	MEDLINE, SCOPUS, and Cochrane up to August 2023	5	RCTs	ACLF	G-CSF	MELD score, CPS	3 were low-risk studies, 2-having some concerns	Administering G-CSF has been shown to positively affect overall survival, liver function, and MELD score	NA
Liu^33^	Cochrane Library, MEDLINE (PubMed), Web of Science, and Ovid EMBASE, Between 2011 and 2021	12	RCT	ACLF	MSCs	MELD score, ALB, coagulation function, survival rate and aminotransferase levels.	One study showed high risk	MSC therapy is a promising option for treating chronic liver disease in clinical settings	Not suspected
Liu^46^	NCBI, Cochrane Library, and MEDLINE up to November 2015	17	Clinical trials	Liver diseases	Hematopoietic stem cells, bone marrow stromal cells	MELD, ALT, AST, TBIL, ALB	Low to moderate	Clinical and biochemical improvements observed in patients with liver diseases following stem cell transplantation suggest that this approach may be a viable clinical solution for treating such individuals	Suspected
Lu^32^	Web of Science, PubMed, EMBASE, and Cochrane Library until May 2023	11	RCTs	Liver cirrhosis	MSCs	ALB, MELD score	High risk-3, 5-low risk, 2-unclear risk studies	MSC therapy improves liver function and protects against complications in liver cirrhosis, without adverse effects	Suspected
Ma^35^	PubMed, EMBASE, and ClinicalTrial.gov, Between January 1990 and June 2014	7	Controlled trials	Liver cirrhosis	AMSCs	MELD score, ALB, TBIL, PT	Low quality	BM-MSC therapy is both safe and efficacious in enhancing liver function	NA
Ouyang^31^	PubMed, Embase, and the Cochrane Library Up to April 2018	9	RCTs and observational studies	Liver cirrhosis	BMSCs	MELD, ALT, TBIL, PT, CPS	NA	treatment with BMDSCs for liver cirrhosis improved short-term MELD and TBIL levels but did not affect mortality risk compared to standard therapy	Not suspected
Pan^29^	Cochrane Library, Med-Line (PubMed), and EMBASE	5	NA	Liver cirrhosis	BM-MSCs	MELD score, ALT, TBIL, PT	NA	BM-MSCs improved liver function in decompensated liver cirrhosis	NA
Pankaj^30^	Medline, EMBASE, PubMed, Science Direct, and the Cochrane Library up to October 2014	8	Case-control studies, cohort, and RCTs	Decompensated liver disease, liver cirrhosis	BM-MSCs	ALB, TBIL, ALT, AST, PT, MELD score, CPS	NA	BM-MSCs demonstrated efficacy in decompensated liver disease	NA
Qiu *et al*.^21^	PubMed, Cochrane Library, and EMBASE Up to March 2023	10	RCTs	ACLF	G-CSF	MELD score, CTP, survival rate, sepsis	Low to moderate risk	G-CSF therapy shows promise as a treatment for ACLF, leading to significant improvements in liver function and survival rates	Suspected
Rajpurohit *et al*.^23^	PubMed, Scopus, and EMBASE	7	RCTS and case-control studies	Liver cirrhosis	G-CSF	MELD score, mortality, disease severity score, change in CD34+ count	Low risk of bias	G-CSF significantly enhances survival rates and disease severity scores while reducing the incidence of complications in patients with liver cirrhosis	NA
Sang^44^	Web of Science, PubMed, EMBASE, Cochrane Library, Wanfang, and CNKI up to April 2017	14	Clinical trials	Liver cirrhosis	UC-MSC	TBIL, ALT, AST, PT, ALB	Moderate	Combining UMSC and TST therapy for LC patients improved liver function, clinical symptoms, and quality of life without severe adverse events, making it a safe and effective therapy for LC.	Suspected
Shi^47^	PubMed, EMBASE, Cochrane Library, and Chinese Biomedical Literature between 2008 and 2020	17	RCTs	Chronic liver disease	G-CSF	MELD score, CPS, survival rate, mortality	Low risk of bias	G-CSF led to significantly improved 12-month survival and reduced Child-Turcotte-Pugh score with relative safety	Unclear
Sun *et al*.^18^	Web of Science, PubMed, Cochrane library, EMBASE, CNKI, VIP, and WanfanguptoMay 2018	10	Clinical trials	HBV-related cirrhosis	ABMSC	MELD, ALT, ALB, AST, TBIL	Moderate risk of bias	ABMSC transplantation via the hepatic artery proved safe and effective in treating HBV-C without inducing severe adverse events.	Not suspected
Tao^45^	Web of Science, PubMed, EMBASE, Cochrane Library, Wanfang and CNKI databaseUptoApril 2017	10	Clinical trials	Liver cirrhosis	UCB SCT	ALT, ALB, AST, TBIL, PT	Low to moderate risk	USCs transplantation is a safe and effective supplementary therapy for LC treated with RST	Suspected
Wang^34^	Cochrane Library, PubMed to April 2022	13	Case-control studies and RCTs	Decompensated liver cirrhosis and ACLF	BMSCsand UC-MSCs	MELD score, TBIL level, ALB level, survival rate, AST coagulation function	Moderate to high quality	Treatment with MSCs is secure and enhances both liver function and survival rates in individuals with end-stage liver disease	NA
Wu^43^	PubMed, Web of Science, CENTRAL, and EBSCO Up to August 15, 2018	15	RCTs and or case–control studies	Liver cirrhosis	ABMSC	AST, TBIL, ALB, PT, CPS, MELD score	NA	Autologous therapy is beneficial for improving liver function and coagulation in patients with liver cirrhosis	NA
Xue^30^	Cochrane Library, MEDLINE, and Embase from inception to November 2017	9	Controlled trials	ACLF	Multipotent cell transplantation	Platelets, hemoglobin, white blood cells, and survival time ALB, PT, ALT, TBIL	2 high quality, 3 moderate, two low, two very low	Transplanting multipotent cells could be suggested as a potential additional therapeutic approach in clinical settings	NA
Xue^42^	Cochrane Library, OVID, EMBASE, and PUBMED up to December 2017	10	RCTS and nonrandomized controlled trials	ACLF	MSCs, BM-MNCs	TBIL, ALT, INR,ALB, MELD score	NA	In the short term, clinical outcomes of stem cell therapy were satisfactory in patients with ACLF. MSCs may offer advantages over BM-MNCs in stem cell transplantation for ACLF	NA
Yang^41^	PubMed, EMBASE, and Web of Science up to February 2015	5	RCTs	Liver failure	G-CSF	PT, TBIL, MELD score, CTPS, survival rate	NA	G-CSF treatment in patients with LF improved liver function, decreased sepsis incidence, and extended short-term survival	Not suspected
Zhao *et al*.^20^	Medline (PubMed), Cochrane Library, EMBASE, ClinicalTrials.gov, and SinoMedCBM Up to June 2017	39	Controlled trials	Liver disease	MSC	MELD score, ALT, ALB, TBIL, PTA/PT	NA	MSC-based therapy is relatively safe and enhances liver function within the initial 6 months post-administration	Suspected
Zhou^38^	MEDLINE, EMBASE, CENTRAL, and ClinicalTrials.gov up to March 16, 2020	24	RCTs	Liver fibrosis, liver cirrhosis, and liver failure	BMSCs, PBSCs, and UC-MSCs	MELD score, TBIL, ALB, ALT, AST, PT, INR, adverse events	High risk	Stem cell therapy is safe and effective for CLD, with patients experiencing improved short-term survival, particularly those with ACLF	Not suspected
Zhu *et al*.^22^	PubMed, Web of Science, EMBASE, and Cochrane Library up to January 2021	8	RCTS and nonrandomized controlled trials	End-stage liver disease	APBSCs, BM-MNCs, HUC-MSCs, ABM-MSCs	MELD score, ALT, ALB, AST, TBIL	Four studies were high risk, two studies were low risk, and one study had a moderate risk of bias	The benefits of combination therapy for enhancing liver function were marginally superior to those of traditional treatment alone, albeit gradually diminishing over time	Not suspected

ACLF, acute-on-chronic liver failure; ALB, albumin; ALT, alanine aminotransferase; APBSCs, allogeneic peripheral blood stem cells; AST, aspartate aminotransferase; BM-MNCs, bone marrow mononuclear cells; BM-MSCs, bone marrow-derived mesenchymal stem cells; CPS, Child–Pugh score; CTP, Child–Turcotte–Pugh score; EBSCO, Elton B. Stephens Company; G-CSF, granulocyte colony-stimulating factor; HBV, hepatitis B virus; IV, intravenous; MELD, Model for End-Stage Liver Disease; MSCs, mesenchymal stem cells; NA, not applicable; PBSCs, peripheral blood stem cells; PT, prothrombin time; RCTs, randomized controlled trials; TBIL, total bilirubin; UC-MSCs, umbilical cord-derived mesenchymal stem cells; UMSCs, umbilical cord mesenchymal stem cells.

### Survival rate

**Figure 2:**
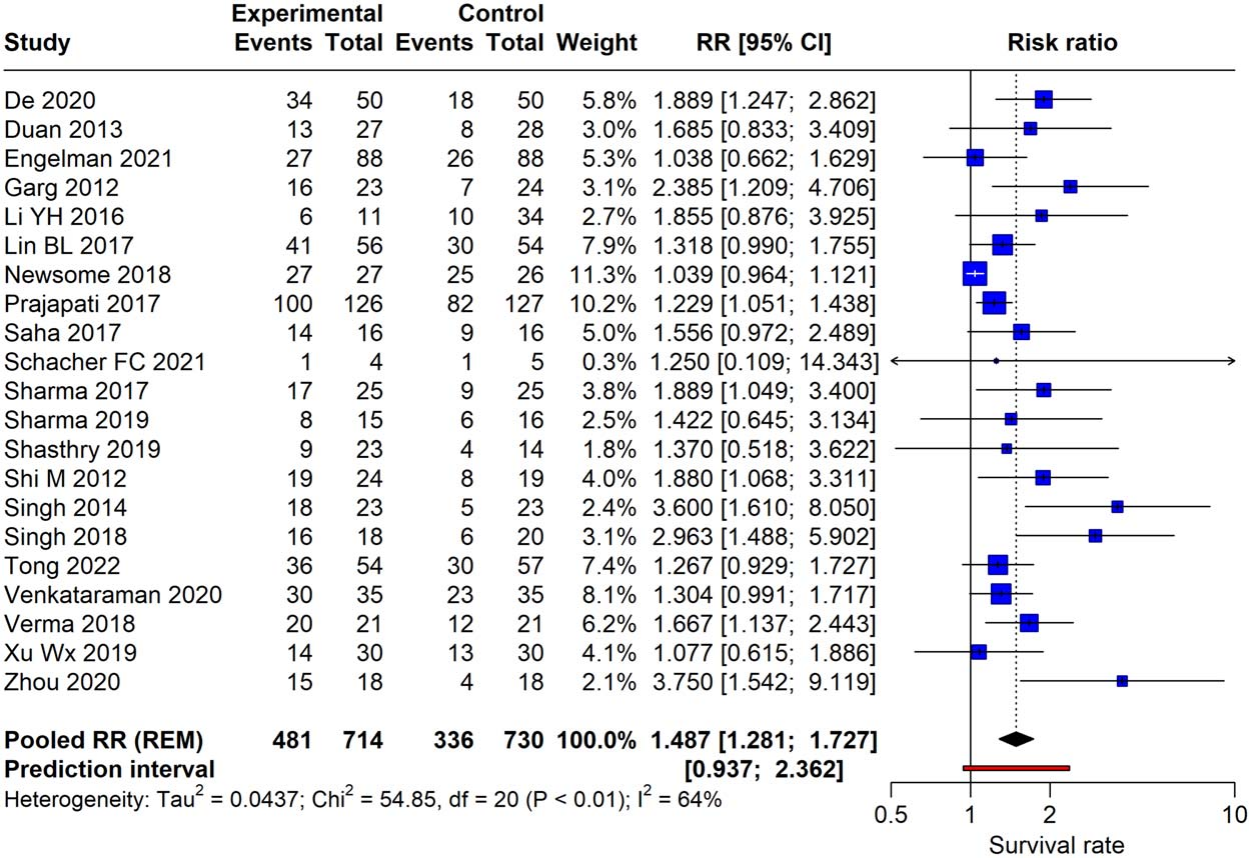
Forest plot depicting survival rate between stem cell therapy and standard care or placebo.

The meta-analysis investigated the survival rate with stem cell therapy in chronic liver disease. From 22 RCTs with a pooled total of 1195 participants, 481 in the experimental groups receiving stem cell therapy and 714 in the control groups, the analysis reveals a pooled RR of 1.487 (95% CI: 1.281–1.727, *P*<0.0001), suggesting a statistically significant improvement in survival rates among those receiving stem cell therapy compared to control groups. The heterogeneity of the included studies, quantified by the *I* ^2^ statistic, was calculated to be 64%, representing a high level of heterogeneity. The forest plot (Fig. 2) graphically displays each study’s weight and effect size, along with the overall pooled effect estimate, illustrating the positive association between stem cell therapy and survival rates in patients with chronic liver disease.

### Albumin levels

**Figure 3:**
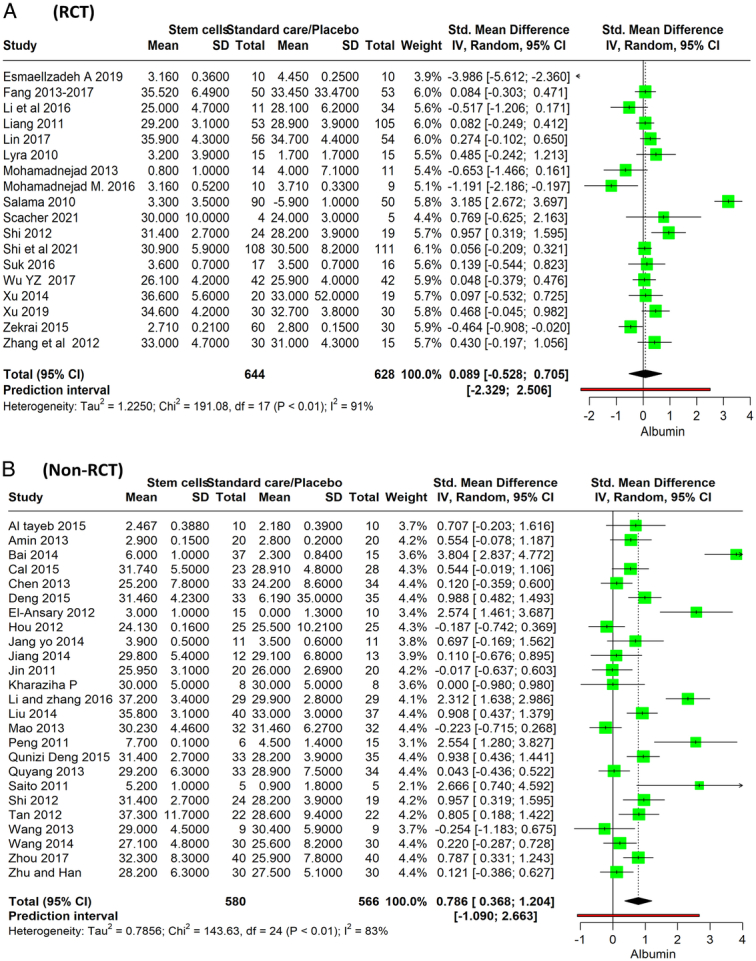
Forest plot depicting albumin level between stem cell therapy and standard care or placebo. (A) RCT, (B) Non-RCT.

Eighteen RCTs with a total of 644 patients receiving stem cell therapy and 528 patients in the standard care or placebo group, indicate a standardized mean difference (SMD) in albumin levels, with the stem cell therapy group showing a slight, but not statistically significant, increase in albumin levels compared to the control group of 0.089 (95% CI: −0.528 to 0.705, *P*=0.765). The heterogeneity of the included studies is high (*I* ^2^=91%; Fig. 3A). The meta-analysis of 24 nonrandomized studies with a combined total of 580 participants in the stem cell therapy groups and 566 participants in the standard care/placebo groups suggests an SMD of 0.786 (95% CI: 0.368–1.204, *P*=0.0007), indicating a positive effect of stem cell therapy on albumin levels compared to the control. The forest plot also indicates a high degree of heterogeneity (*I* ^2^=82%; Fig. 3B).

### Model for End-Stage Liver Disease score

**Figure 4:**
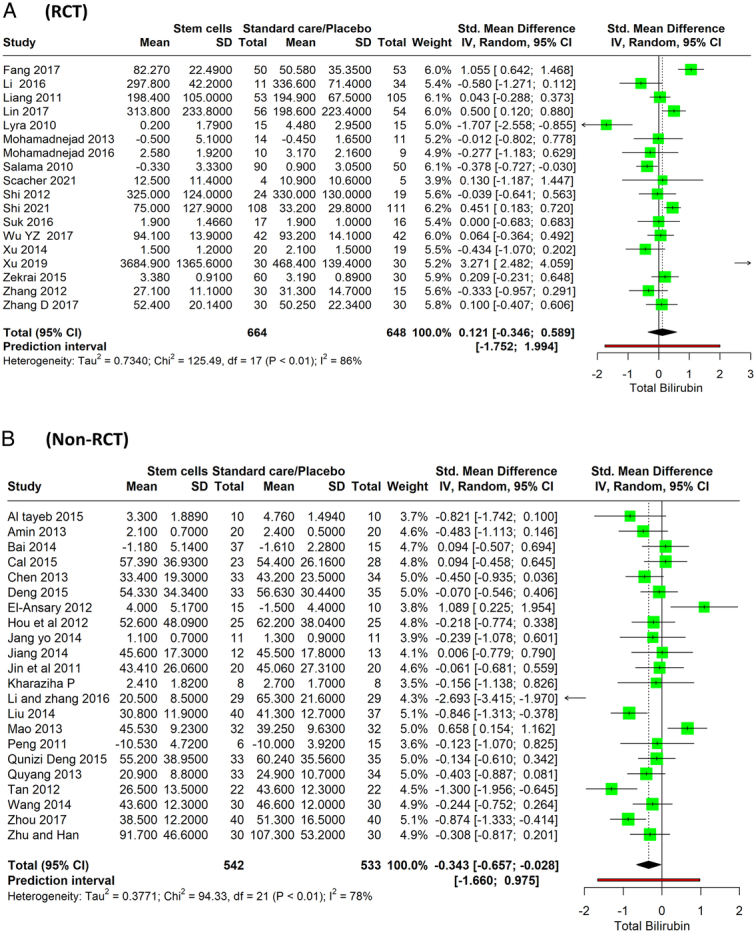
Forest plot depicting total bilirubin level between stem cell therapy and standard care or placebo. (A) RCT, (B) Non-RCT.

The meta-analysis encompasses 22 RCTs, including a total of 677 patients who received stem cell therapy and 675 who received standard care or a placebo, and showed a pooled estimate of the SMD −0.484 (95% CI: −0.900 to −0.068, *P*=0.0248), suggesting that stem cell therapy is associated with a statistically significant reduction in MELD score compared to standard care or placebo. The negative sign indicates that stem cell therapy may lead to an improvement, as lower MELD scores are associated with better liver function. There is a high degree of heterogeneity (*I* ^2^=92%) (Fig. 4A). The analysis from 10 nonrandomized studies, which altogether encompass 198 patients in the stem cell therapy groups and 219 in the standard care/placebo groups, showed SMD of −0.171 (95% CI: −0.377 to 0.034, *P*=0.091). The CI suggests that there is uncertainty about the precise magnitude of the effect, with the possibility of a slight reduction or a slight increase in MELD scores due to stem cell therapy. There is little to no heterogeneity among the results of the included studies (*I* ^2^=0%), implying consistency in the findings across these studies (Fig. 4B).

### Total bilirubin

**Figure 5:**
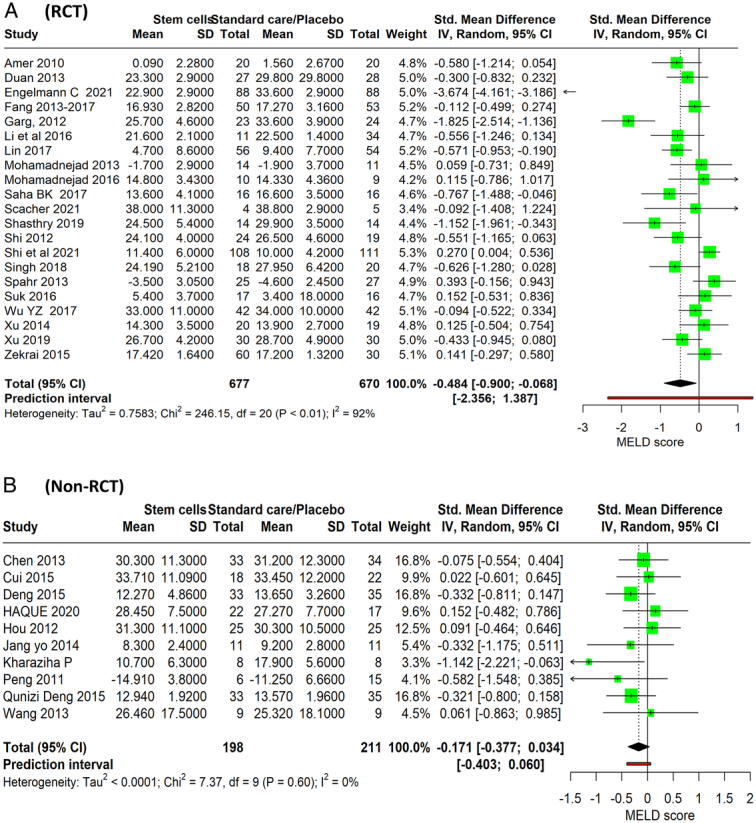
Forest plot depicting MELD scores between stem cell therapy and standard care or placebo. MELD, Model for End-Stage Liver Disease. (A) RCT, (B) Non-RCT.

From the analysis, 18 RCTs are included, with a total of 664 patients receiving stem cell therapy and 648 patients receiving standard care or a placebo. The pooled SMD across these studies is 0.121 (95% CI: −0.346 to 0.589, *P*=0.591), indicating that there is no significant difference in total bilirubin levels between the stem cell therapy group and the standard care or placebo group. The CI crosses 0, meaning that the impact of stem cell treatment on total bilirubin is not statistically significant. There is high heterogeneity in the results (*I* ^2^=86%) (Fig. 5A). The combined results from 21 nonrandomized studies involving 542 participants in the stem cell therapy groups and 533 participants in the standard care/placebo groups indicate an SMD of −0.343 (95% CI: −0.657 to −0.028, *P*=0.034). This points to a lower mean total bilirubin level in the stem cell therapy groups compared to standard care/placebo, suggesting a potential beneficial effect of the therapy. The analysis demonstrated considerable heterogeneity among the studies, with an *I* ^2^ value of 78% (Fig. 5B).

### Alanine transaminase

**Figure 6:**
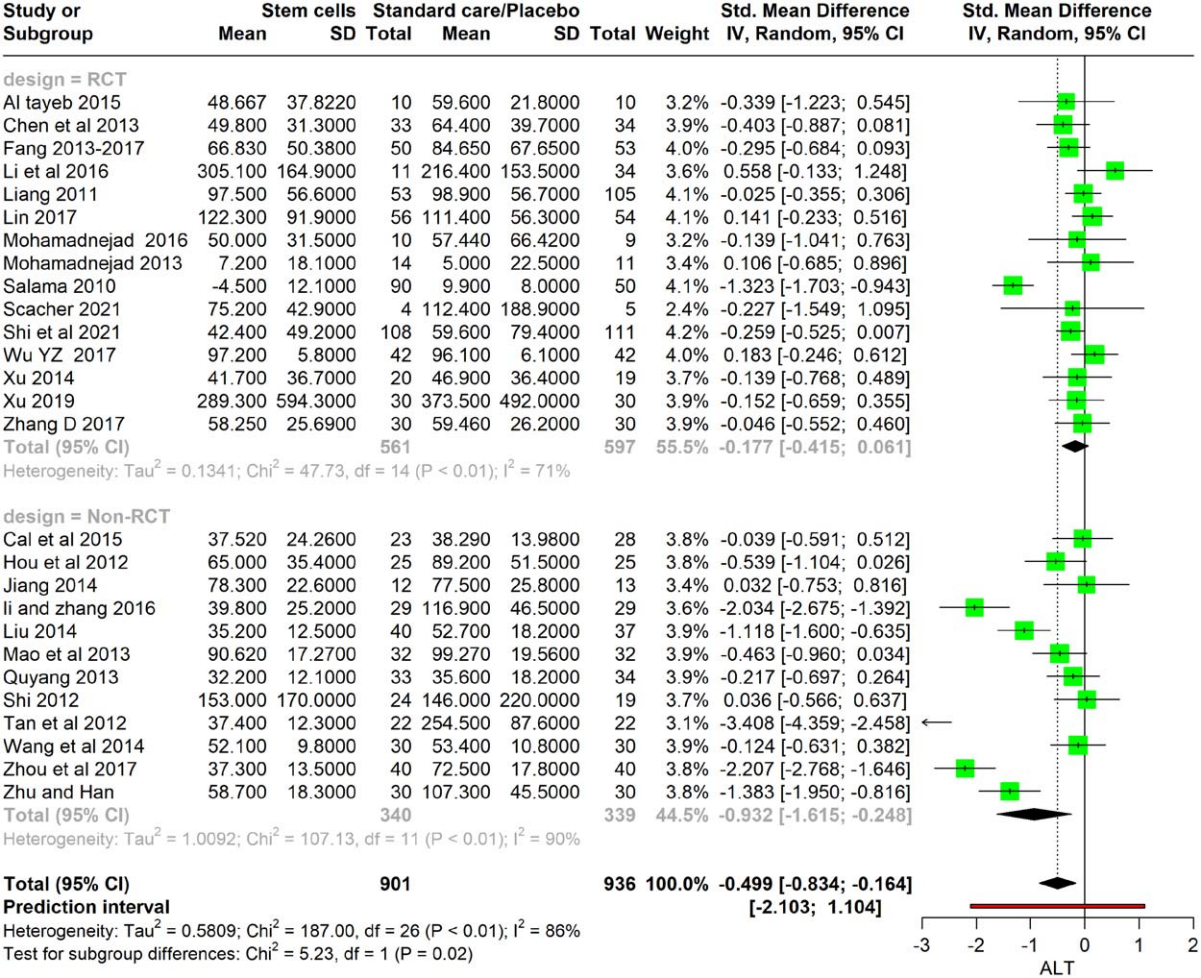
Forest plot depicting ALT level between stem cell therapy and standard care or placebo. ALT, alanine aminotransferase.

The analysis of a total of 14 RCTs with 561 participants and 13 non-RCTs with 340 participants. Results from the RCT subgroup show a nonsignificant SMD of −0.177 (95% CI: −0.415 to 0.061), while the non-RCT subgroup displays a significantly larger SMD of −0.932 (95% CI: −1.615 to −0.248). The heterogeneity within these subgroups is substantial, with *I* ^2^ values of 71% for RCTs and 90% for non-RCTs, indicating notable variability in the outcomes across studies. Overall, the meta-analysis yields an SMD of −0.499 (95% CI: −0.834 to −0.164), signaling a significant decrease in ALT levels attributable to stem cell therapy when considering all studies (Fig. 6).

### Aspartate aminotransferase

**Figure 7:**
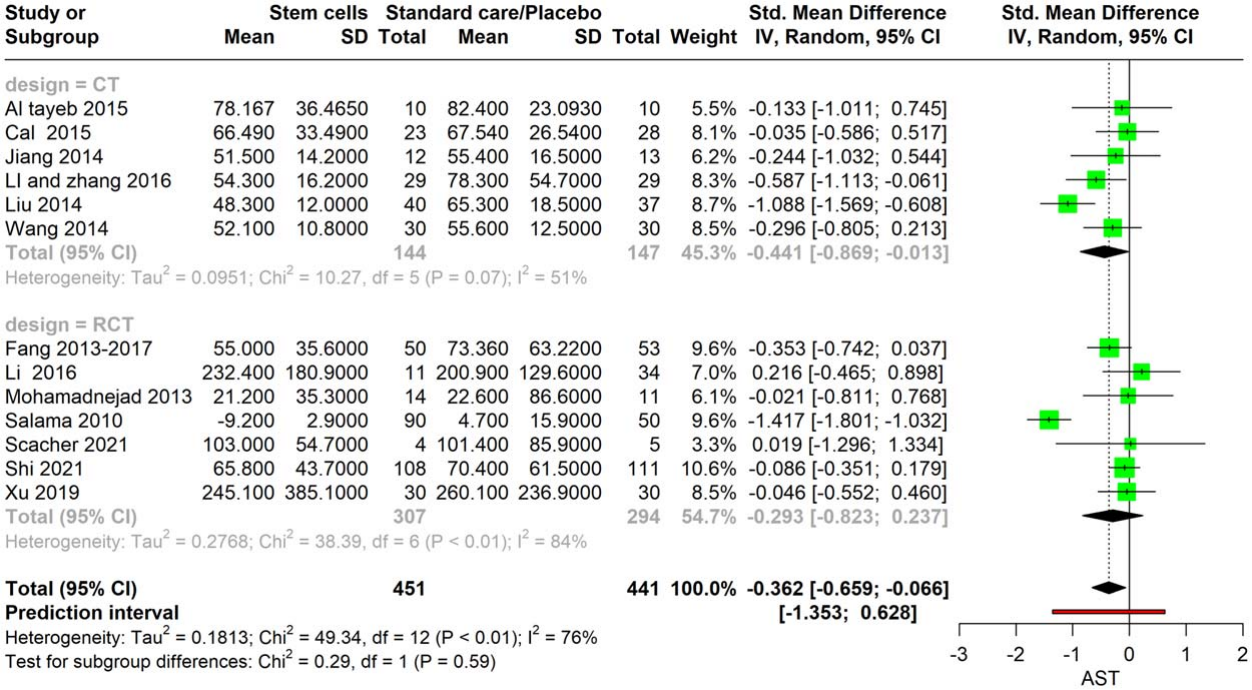
Forest plot depicting AST level between stem cell therapy and standard care or placebo. AST, aspartate aminotransferase.

This meta-analysis evaluates the efficacy of stem cell therapy in altering AST levels, examining both controlled trials (CTs) and RCTs. In total, the analysis comprises 451 participants across the two groups: 144 participants in six CTs and 307 participants in seven RCTs. The CT subgroup showed an SMD of −0.441 (95% CI: −0.869 to −0.013), suggesting a small but significant decrease in AST levels with stem cell therapy. However, the heterogeneity within this subgroup was low (*I* ^2^=51%). Conversely, the RCT subgroup had a slightly higher SMD of −0.293 (95% CI: −0.823 to 0.237), indicating a reduction in AST levels, although this did not reach statistical significance, which could be reflective of the higher heterogeneity present (*I* ^2^ = 84%). Combining the data from both CTs and RCTs yields an overall SMD of −0.362 (95% CI: −0.659 to −0.066), with the CI suggesting a statistically significant reduction in AST levels due to stem cell therapy. However, the total heterogeneity for the combined analysis is considerable (*I* ^2^=76%), which indicates substantial variability in the stem cell treatment on AST levels among the included studies (Fig. 7).

### Publication bias

Funnel plots and the Egger test were used to determine the presence of publication bias for each outcome. Survival rate (Egger, *P*<0.0001) and albumin levels from nonrandomized studies (Egger, *P*=0.0249) showed possible presence of publication bias. For the rest of the outcomes, publication bias is not suspected according to statistical analysis.

## Discussion

The findings from this umbrella review provide an overview of the efficacy of stem cell therapy in patients with chronic liver diseases, with a particular focus on liver cirrhosis. Stem cell therapy has been posited as a novel therapeutic approach with the potential to address the limitations of conventional treatments for liver cirrhosis. This review synthesized evidence from multiple systematic reviews and meta-analyses, offering insights into the impact of stem cell therapy on liver function and survival rates in individuals with liver cirrhosis and other chronic liver diseases.

One of the most significant findings of our analysis was the improvement in survival rates among patients receiving stem cell therapy compared to those in control groups. The pooled RR of 1.487 indicates a statistically significant improvement, albeit with a high level of heterogeneity among the included studies. This heterogeneity may be attributed to differences in study designs, stem cell sources, and patient populations across the studies. Despite this, the overall positive trend in survival rates is encouraging and suggests that stem cell therapy could offer a meaningful benefit to patients with chronic liver diseases. This finding aligns with the regenerative potential of MSCs, which, through their immunomodulatory and anti-fibrotic effects, could contribute to improved liver function and, consequently, survival outcomes. As indicated by albumin levels and the MELD score, stem cell therapy’s impact on liver function presents a more comprehensive picture. While the analysis of nonrandomized studies suggested a positive effect of stem cell therapy on albumin levels, RCTs did not show a statistically significant difference. This discrepancy underscores the complexity of assessing therapeutic outcomes in liver cirrhosis. It highlights the need for well-designed, large-scale RCTs to elucidate better the effects of stem cell therapy on liver function. Regarding liver biochemistry, our review found no significant difference in total bilirubin between stem cell therapy and control groups in RCTs, whereas nonrandomized studies suggested a potentially beneficial effect. Total bilirubin, a key marker of liver function, and its levels not significantly changing could indicate that while stem cell therapy has promising effects on survival and MELD scores, its impact on all aspects of liver biochemistry requires further investigation. The analysis of ALT and AST levels, enzymes indicative of liver injury, showed a significant decrease in levels attributable to stem cell therapy, particularly in non-RCTs. These findings suggest stem cell therapy may have hepatoprotective effects, reducing liver inflammation and injury. Nonetheless, the substantial heterogeneity and the more pronounced effects observed in nonrandomized studies highlight the need for more rigorous RCTs to confirm these potential benefits.

The call for standardization in stem cell therapy protocols is of paramount importance. The variability in cell sources ranging from bone marrow-derived MSCs to umbilical cord-derived MSCs and adipose tissue-derived stem cell preparation methods and dosing regimens currently represents a significant challenge in comparing and consolidating results across studies. Establishing consensus guidelines on these aspects would not only facilitate more uniform research practices but also enhance the reproducibility and comparability of findings, thereby providing clearer insights into the therapy’s efficacy and safety^55^. In addition to standardization, the emphasis on conducting more high-quality RCTs cannot be overstated. The current evidence landscape is marked by a mix of promising results and significant variability, partly due to the methodological differences and limitations of existing studies. Future RCTs should be designed with sufficient sample sizes to achieve the necessary power to detect clinically meaningful effects, rigorous control groups, and standardized outcome measures. These studies should not only assess the short-term efficacy and safety of stem cell therapy but also include long-term follow-up periods to evaluate the durability of treatment effects, potential late adverse events, and overall survival benefits. Understanding the mechanisms of action through which stem cells exert their therapeutic effects on the liver is crucial. This includes delving deeper into their immunomodulatory functions, anti-fibrotic effects, and abilities to promote regeneration of liver tissue^56,57^. Advanced techniques in molecular biology and bioinformatics could be leveraged to explore these mechanisms at the cellular and molecular levels, potentially identifying specific pathways that are modulated by stem cell therapy. Such insights could lead to the identification of biomarkers for response to therapy, enabling personalized treatment approaches and optimization of treatment strategies. The exploration of combinational therapies deserves attention. Given the complex pathophysiology of chronic liver diseases, a multifaceted treatment approach may be more effective. Investigating the potential synergies between stem cell therapy and other therapeutic modalities, such as pharmacological treatments, lifestyle interventions, and other regenerative medicine strategies, could open new avenues for comprehensive treatment regimens^58^.

Stem cell therapy, emerging as a promising novel therapeutic approach, offers hope beyond the limitations of current conventional treatments. This section delineates the potential clinical implications, emphasizing how these insights could guide future therapeutic strategies and clinical practice. The positive trend observed in survival rates among patients receiving stem cell therapy suggests that this approach could serve as a valuable addition to the arsenal of treatments for chronic liver diseases^59^. Clinicians may consider stem cell therapy as a complementary treatment to enhance patient outcomes, particularly in cases where conventional treatments have failed to yield satisfactory results or are not feasible due to limitations such as the scarcity of donor organs for transplantation. The heterogeneity noted in the outcomes across different studies points to the need for a personalized medicine approach in administering stem cell therapy. By understanding patient-specific variables, such as the underlying cause of liver disease, the stage of liver cirrhosis, and individual patient response to stem cell therapy, clinicians can tailor treatment plans to maximize efficacy and minimize risks. The identification of biomarkers for response to stem cell therapy, as suggested by future research directions, would further facilitate this personalized approach^60^. The potential benefits of stem cell therapy in improving liver function, reducing liver inflammation and injury, and improving survival rates underscore its role within a multidisciplinary care framework. Clinicians, including hepatologists, transplant surgeons, and regenerative medicine specialists, should engage in multidisciplinary discussions to integrate stem cell therapy into the comprehensive care plan for patients with chronic liver diseases. This collaborative approach ensures that all therapeutic options are considered and that patients receive holistic, patient-centered care. Given the innovative nature of stem cell therapy and the complexities involved in its application, educating patients and their caregivers about the potential benefits, risks, and uncertainties of this treatment is crucial. Informed consent processes should encompass detailed discussions about the current evidence, the experimental nature of many stem cell therapies, and the ongoing need for research to fully understand the long-term implications of this treatment option.

Our study has a few limitations. One notable limitation is the inherent heterogeneity among the included studies regarding study designs, stem cell sources, preparation methods, dosing regimens, and patient populations. This diversity makes it challenging to draw definitive conclusions about the efficacy and safety of stem cell therapy across the board. Furthermore, depending on published literature could lead to publication bias since studies reporting positive outcomes are often more frequently published compared to those with negative or inconclusive findings. Additionally, the exclusion of non-English articles and unpublished studies could have omitted relevant data, potentially affecting the review’s comprehensiveness. Finally, the rapidly evolving nature of stem cell research means that newer studies may have been published after our search date and not included in this review, which could affect the currency and relevance of our findings. These limitations emphasize the importance of careful interpretation of the findings and the need for high-quality, standardized research in the field of stem cell therapy for chronic liver diseases.

## Conclusion

Our analysis offers valuable insights into the potential of stem cell treatment as a novel approach for treating chronic liver diseases. The observed improvement in survival rates and certain liver function markers indicates that stem cell therapy can be a potential option for liver disease. However, ambiguity still exists in fully understanding the beneficial effects of stem cell therapy. Future efforts must focus on standardizing therapy protocols and conducting high-quality RCTs to elucidate stem cell therapy’s efficacy and safety fully. Advancing stem cell treatment for chronic liver diseases requires a careful balance between innovative treatment strategies and the rigor of scientific validation to ensure patient safety and optimal clinical outcomes.

## Ethics approval and consent to participate

Not applicable.

## Consent for publication

All authors gave consent for publication.

## Sources of funding

Not applicable.

## Author contribution

Y.T., M.N.K., S.A., A.A.Z., Q.S.Z.: conceptualization. A.M.G., A.A., R.K.S.: data curation. H.F.A., Q.S.Z., S.R.: formal analysis. M.A.A., S.A.A., A.A.: investigation. A.M., Q.S.Z., M.N.K.: methodology. S.A., P.S.: project administration. P.S., R.K.S.: resources. A.M., B.C.S., B.K.P.: software. A.H., S.R., A.A.R., B.C.S., F.A.M.A.: supervision. A.M., R.A.A., R.A., B.K.P.: validation. A.M.G., F.A.M.A., S.A.A.: visualization.

## Conflicts of interest disclosure

The authors declares no conflicts of interest.

## Research registration unique identifying number (UIN)

PROSPERO: CRD42024507255.

## Guarantor

Bijaya K. Padhi.

## Availability of data and materials

All data are presented within the manuscript and are available by contacting the corresponding author.

## Provenance and peer review

Not applicable.

## Acknowledgements

The authors acknowledge Nested Knowledge, MN, USA, for providing access to the software.

## Supplementary Material
