# Supplementary material for: Efficacy of stem cell therapy in patients with chronic liver disease: an umbrella review of systematic reviews
Source: Int J Surg. 2024 May 22;110(11):6848–61. doi: 10.1097/JS9.0000000000001644 (PMC11573100; doi:10.1097/JS9.0000000000001644)
Supplement: Supplementary file 1 [file js9-110-6848-s001.docx]

**SUPPLEMENTARY MATERIALS**

## **Table S1.** PRISMA Checklist

| **Section and Topic** | **Item #** | **Checklist item** | **Location where item is reported** |
| --- | --- | --- | --- |
| **TITLE** | | |  |
| Title | 1 | Identify the report as a systematic review. | 1 |
| **ABSTRACT** | | |  |
| Abstract | 2 | See the PRISMA 2020 for Abstracts checklist. (made as per the Journal guidelines) | 2 |
| **INTRODUCTION** | | |  |
| Rationale | 3 | Describe the rationale for the review in the context of existing knowledge. | 3 |
| Objectives | 4 | Provide an explicit statement of the objective(s) or question(s) the review addresses. | 3 |
| **METHODS** | | |  |
| Eligibility criteria | 5 | Specify the inclusion and exclusion criteria for the review and how studies were grouped for the syntheses. | 4 |
| Information sources | 6 | Specify all databases, registers, websites, organisations, reference lists and other sources searched or consulted to identify studies. Specify the date when each source was last searched or consulted. | 4 |
| Search strategy | 7 | Present the full search strategies for all databases, registers and websites, including any filters and limits used. | Table S3 |
| Selection process | 8 | Specify the methods used to decide whether a study met the inclusion criteria of the review, including how many reviewers screened each record and each report retrieved, whether they worked independently, and if applicable, details of automation tools used in the process. | 4 |
| Data collection process | 9 | Specify the methods used to collect data from reports, including how many reviewers collected data from each report, whether they worked independently, any processes for obtaining or confirming data from study investigators, and if applicable, details of automation tools used in the process. | 4, 5 |
| Data items | 10a | List and define all outcomes for which data were sought. Specify whether all results that were compatible with each outcome domain in each study were sought (e.g., for all measures, time points, analyses), and if not, the methods used to decide which results to collect. | 4, Table 1 |
|  | 10b | List and define all other variables for which data were sought (e.g., participant and intervention characteristics, funding sources). Describe any assumptions made about any missing or unclear information. | 4 |
| Study risk of bias assessment | 11 | Specify the methods used to assess risk of bias in the included studies, including details of the tool(s) used, how many reviewers assessed each study and whether they worked independently, and if applicable, details of automation tools used in the process. | 5, Table S4 |
| Effect measures | 12 | Specify for each outcome the effect measure(s) (e.g. risk ratio, mean difference) used in the synthesis or presentation of results. | 5 |
| Synthesis methods | 13a | Describe the processes used to decide which studies were eligible for each synthesis (e.g. tabulating the study intervention characteristics and comparing against the planned groups for each synthesis (item #5)). | 5, Table 1 |
|  | 13b | Describe any methods required to prepare the data for presentation or synthesis, such as handling of missing summary statistics, or data conversions. | NA |
|  | 13c | Describe any methods used to tabulate or visually display results of individual studies and syntheses. | 4 |
|  | 13d | Describe any methods used to synthesize results and provide a rationale for the choice(s). If meta-analysis was performed, describe the model(s), method(s) to identify the presence and extent of statistical heterogeneity, and software package(s) used. | 5 |
|  | 13e | Describe any methods used to explore possible causes of heterogeneity among study results (e.g. subgroup analysis, meta-regression). | 6 |
|  | 13f | Describe any sensitivity analyses conducted to assess robustness of the synthesized results. | 6 |
| Reporting bias assessment | 14 | Describe any methods used to assess risk of bias due to missing results in a synthesis (arising from reporting biases). | NA |
| Certainty assessment | 15 | Describe any methods used to assess certainty (or confidence) in the body of evidence for an outcome. | NA |
| **RESULTS** | | |  |
| Study selection | 16a | Describe the results of the search and selection process, from the number of records identified in the search to the number of studies included in the review, ideally using a flow diagram. | Figure-1 |
|  | 16b | Cite studies that might appear to meet the inclusion criteria, but which were excluded, and explain why they were excluded. | 6, Table 1 |
| Study characteristics | 17 | Cite each included study and present its characteristics. | Table-1 |
| Risk of bias in studies | 18 | Present assessments of risk of bias for each included study. | Table S4 |
| Results of individual studies | 19 | For all outcomes, present, for each study: (a) summary statistics for each group (where appropriate) and (b) an effect estimate and its precision (e.g. confidence/credible interval), ideally using structured tables or plots. | Table 1, Figure 2,3 |
| Results of syntheses | 20a | For each synthesis, briefly summarise the characteristics and risk of bias among contributing studies. | 5 |
|  | 20b | Present results of all statistical syntheses conducted. If meta-analysis was done, present for each the summary estimate and its precision (e.g. confidence/credible interval) and measures of statistical heterogeneity. If comparing groups, describe the direction of the effect. | 5, 6 Figure 2, 3 |
|  | 20c | Present results of all investigations of possible causes of heterogeneity among study results. | 6 |
|  | 20d | Present results of all sensitivity analyses conducted to assess the robustness of the synthesized results. | NA |
| Reporting biases | 21 | Present assessments of risk of bias due to missing results (arising from reporting biases) for each synthesis assessed. | NA |
| Certainty of evidence | 22 | Present assessments of certainty (or confidence) in the body of evidence for each outcome assessed. | NA |
| **DISCUSSION** | | |  |
| Discussion | 23a | Provide a general interpretation of the results in the context of other evidence. | 6 |
|  | 23b | Discuss any limitations of the evidence included in the review. | 7 |
|  | 23c | Discuss any limitations of the review processes used. | 8 |
|  | 23d | Discuss implications of the results for practice, policy, and future research. | 9 |
| **OTHER INFORMATION** | | |  |
| Registration and protocol | 24a | Provide registration information for the review, including register name and registration number, or state that the review was not registered. | 4 |
|  | 24b | Indicate where the review protocol can be accessed, or state that a protocol was not prepared. | 4 |
|  | 24c | Describe and explain any amendments to information provided at registration or in the protocol. | NA |
| Support | 25 | Describe sources of financial or non-financial support for the review, and the role of the funders or sponsors in the review. | 9 |
| Competing interests | 26 | Declare any competing interests of review authors. | 9 |
| Availability of data, code and other materials | 27 | Report which of the following are publicly available and where they can be found: template data collection forms; data extracted from included studies; data used for all analyses; analytic code; any other materials used in the review. | 9 |

**Table S2**: Search strategy

| Database | No | Search Query | Results |
| --- | --- | --- | --- |
| Date:29-02-2024 | |  | |
| PubMed/ OVID-Medline | **#1** | ((((((Chronic liver failure[Title/Abstract]) ) OR (liver failure[Title/Abstract])) OR (hepatic failure[Title/Abstract])) OR (liver cirrhosis[Title/Abstract])) OR (hepatic cirrhosis[Title/Abstract])) OR (cirrhosis[Title/Abstract]) | 139183 |
|  | **#2** | ((((((((colony forming units[Title/Abstract]) OR (stem cells[Title/Abstract])) OR (stromal cells[Title/Abstract])) OR (wharton jelly cells[Title/Abstract])) OR (mother cell[Title/Abstract])) OR (mesenchymal stem cell[Title/Abstract])) OR (progenitor cells[Title/Abstract])) OR (bone marrow[Title/Abstract])) OR (umbilical cord[Title/Abstract]) | 538827 |
|  | **#3** | (systematic review[Title]/Abstract) OR (meta- analysis[Title/Abstract]) | 418101 |
|  | **#4** | #1AND #2AND#3 | 37 |
| EMBASE | **#1** | 'stem cells':ti,ab,kw OR 'stromal cells':ti,ab,kw OR 'wharton jelly cells':ti,ab,kw OR 'mother cell':ti,ab,kw OR 'mesenchymal stem cell':ti,ab,kw OR 'progenitor cells':ti,ab,kw OR 'bone marrow':ti,ab,kw OR 'umbilical cord':ti,ab,kw OR 'colony forming units':ti,ab,kw | 770266 |
|  | **#2** | 'Chronic liver failure':ti,ab,kw OR ' liver cirrhosis’:ti,ab,kw OR ‘liver failure’:ti,ab,kw OR 'cirrhosis’:ti,ab,kw OR 'hepatic failure’:ti,ab,kw OR 'hepatic cirrhosis':ti,ab,kw OR 'chronic hepatic failure':ti,ab,kw | 224454 |
|  | **#3** | 'systematic review':ti,ab OR 'meta-analysis':ti,ab | 508643 |
|  | **#4** | #1 AND #2AND #3 | 54 |
| WOS  advanced | **#1** | TS=((“stem cells” OR “stromal cells” OR “Wharton’s Jelly cells” OR “mother cell” OR "mesenchymal stem cell" OR “progenitor cells” OR “bone marrow” OR “umbilical cord” OR “colony forming units”) ) | 703418 |
|  | **#2** | TS=((“chronic liver failure” OR “liver cirrhosis” OR “liver failure” OR cirrhosis OR “hepatic failure” OR “hepatic cirrhosis” OR “chronic hepatic failure”)) | 140890 |
|  | **#3** | **(((TI=(("Systematic review" or "meta-analysis")))) OR AB=(systematic review)) OR AB=(meta-analysis)** | 488870 |
|  | **#4** | #1 AND #2AND #3 | 66 |
| Cochrane | **#1** | ((“stem cells” OR “stromal cells” OR “Wharton’s Jelly cells” OR “mother cell” OR "mesenchymal stem cell" OR “progenitor cells” OR “bone marrow” OR “umbilical cord” OR “colony forming units”)):ti,ab,kw | 35151 |
|  | **#2** | ((“chronic liver failure” OR “liver cirrhosis” OR “liver failure” OR cirrhosis OR “hepatic failure” OR “hepatic cirrhosis” OR “chronic hepatic failure”)):ti,ab,kw" | 13954 |
|  | **#3** | ("Systematic review" or "meta-analysis"):ti,ab,kw” | 26958 |
|  | **#4** | #1 AND #2AND #3 | 5 |
|  |  |  |  |

**Table S3.** Summary of quality assessment of included systematic reviews using AMSTAR2

| Study ID | 1 | 2 | 3 | 4 | 5 | 6 | 7 | 8 | 9 | 10 | 11 | 12 | 13 | 14 | 15 | 16 |  |
| --- | --- | --- | --- | --- | --- | --- | --- | --- | --- | --- | --- | --- | --- | --- | --- | --- | --- |
|  | **n-critical domain** | **Critical domain** | **n-critical domain** | **Critical domain** | **n-critical domain** | **n-critical domain** | **Critical domain** | **n-critical domain** | **Critical domain** | **n-critical domain** | **Critical domain** | **n-critical domain** | **Critical domain** | **n-critical domain** | **Critical domain** | **n-critical domain** | **Overall** |
|  | **PICO** | **PROSPERO protocol** | **selection of the study designs for inclusion** | **search strategy** | **study selection in duplicate** | **data extraction in duplicate?** | **list of excluded studies and justify the exclusions?** | **authors describe the included studies in adequate detail?** | **risk of bias (RoB) in individual studies** | **Did the review authors report on the sources of funding for the studies included in the review?** | **If meta-analysis was performed did the review authors use appropriate methods for statistical combination of results?** | **If meta-analysis was performed, did the review authors assess the potential impact of RoB in individual studies on the results of the**  **meta-analysis or other evidence synthesis?** | **authors account for RoB in individual studies when interpreting/ discussing the results** | **Did the review authors provide a satisfactory explanation for, and discussion of, any heterogeneity observed in the results of the review?** | **publication bias (small study bias)** | **conflict of interest, including any funding they received** |  |
| AdiwinataPawitan 2019 [1] | Yes | Yes | Yes | Yes | Yes | Yes | No | Yes | Yes | No | Yes | Yes | No | No | Yes | Yes | Moderate |
| Chavez-Tapia 2015 [2] | Yes | Yes | Yes | Yes | No | No | No | Yes | no | No | Yes | Yes | No | No | No | Yes | Low |
| Chen 2018 [3] | Yes | Yes | Yes | Yes | Yes | Yes | No | Yes | Yes | No | Yes | Yes | Yes | no | No | Yes | Low |
| Chen 2019 [4] | Yes | Yes | Yes | Yes | No | No | No | Yes | Yes | No | Yes | Yes | no | no | No | Yes | Critically low |
| Huang 2021 [5] | Yes | Yes | Yes | Yes | Yes | Yes | No | Yes | No | No | Yes | Yes | No | No | Yes | Yes | Low |
| Kim 2015 [6] | Yes | Yes | Yes | Yes | Yes | Yes | No | no | Yes | No | No | No | No | No | No | Yes | Critically low |
| Konstantis 2023 [7] | Yes | Yes | Yes | Yes | Yes | Yes | No | Yes | No | No | No | No | Yes | no | No | Yes | Critically low |
| Liu 2023 [8] | Yes | Yes | Yes | Yes | No | No | No | Yes | Yes | No | No | No | yes | No | No | Yes |  |
| Liu 2016 [9] | Yes | No | Yes | Yes | Yes | Yes | No | Yes | Yes | No | Yes | Yes | Yes | No | No | Yes | Low |
| Lu 2023 [10] | Yes | Yes | Yes | Yes | Yes | Yes | No | Yes | Yes | No | Yes | Yes | No | No | Yes | Yes | Moderate |
| Ma 2015 [11] | Yes | No | Yes | Yes | Yes | Yes | No | Yes | no | No | Yes | Yes | No | No | No | Yes | Low |
| Ouyang 2021 [12] | Yes | No | Yes | Yes | No | No | No | Yes | No | No | Yes | Yes | No | No | No | Yes | Low |
| Pan 2014 [13] | Yes | No | Yes | Yes | Yes | Yes | No | Yes | Yes | No | Yes | Yes | No | No | No | Yes | Moderate |
| Pankaj 2015 [14] | Yes | Yes | Yes | Yes | Yes | Yes | No | Yes | Yes | No | Yes | Yes | No | No | No | Yes | Low |
| Qiu 2023 [15] | Yes | Yes | Yes | Yes | No | No | No | Yes | Yes | No | No | No | No | No | No | Yes | Critically low |
| Rajpurohit 2023 [16] | Yes | no | Yes | Yes | Yes | Yes | No | Yes | Yes | No | Yes | yes | No | No | Yes | Yes | Low |
| Sang 2018 [17] | Yes | No | Yes | Yes | Yes | Yes | No | Yes | Yes | No | Yes | No | No | No | No | Yes | Critically low |
| Shi 2022 [18] | Yes | Yes | Yes | Yes | Yes | Yes | No | Yes | Yes | No | Yes | No | No | No | Yes | Yes | Low |
| Sun 2020 [19] | Yes | Yes | Yes | Yes | Yes | Yes | No | Yes | Yes | No | Yes | No | Yes | no | Yes | Yes | Moderate |
| Tao 2018 [20] | Yes | Yes | Yes | Yes | Yes | Yes | No | Yes | Yes | No | Yes | No | no | no | Yes | Yes | Low |
| Wang 2023 [21] | Yes | No | Yes | Yes |  |  | No | Yes | Yes | No | Yes | No | No | No | Yes | Yes | Low |
| Wu 2019 [22] | Yes | Yes | Yes | Yes | No | No | No | Yes | No | No | Yes | No | No | No | No | Yes | Critically low |
| Xue 2018 [23] | Yes | Yes | Yes | Yes | No | No | No | Yes | Yes | No | Yes | No | Yes | no | Yes | Yes | Low |
| Xue 2018 [24] | Yes | No | Yes | Yes | No | No | No | Yes | Yes | No | Yes | No | yes | No | Yes | Yes | Low |
| Yang 2016 [25] | Yes | No | Yes | Yes | No | No | No | Yes | No | No | Yes | Yes | No | No | No | Yes | Low |
| Zhao 2018 [26] | Yes | No | Yes | Yes | Yes | Yes | No | Yes | Yes | No | Yes | Yes | No | No | No | Yes | Moderate |
| Zhou 2020 [27] | Yes | Yes | Yes | Yes | Yes | Yes | No | Yes | Yes | No | Yes | Yes | No | No | No | Yes | Low |
| Zhu 2021 [28] | Yes | no | Yes | Yes | Yes | Yes | No | Yes | Yes | No | Yes | yes | No | No | Yes | Yes | Low |

**Funnel plots**

**
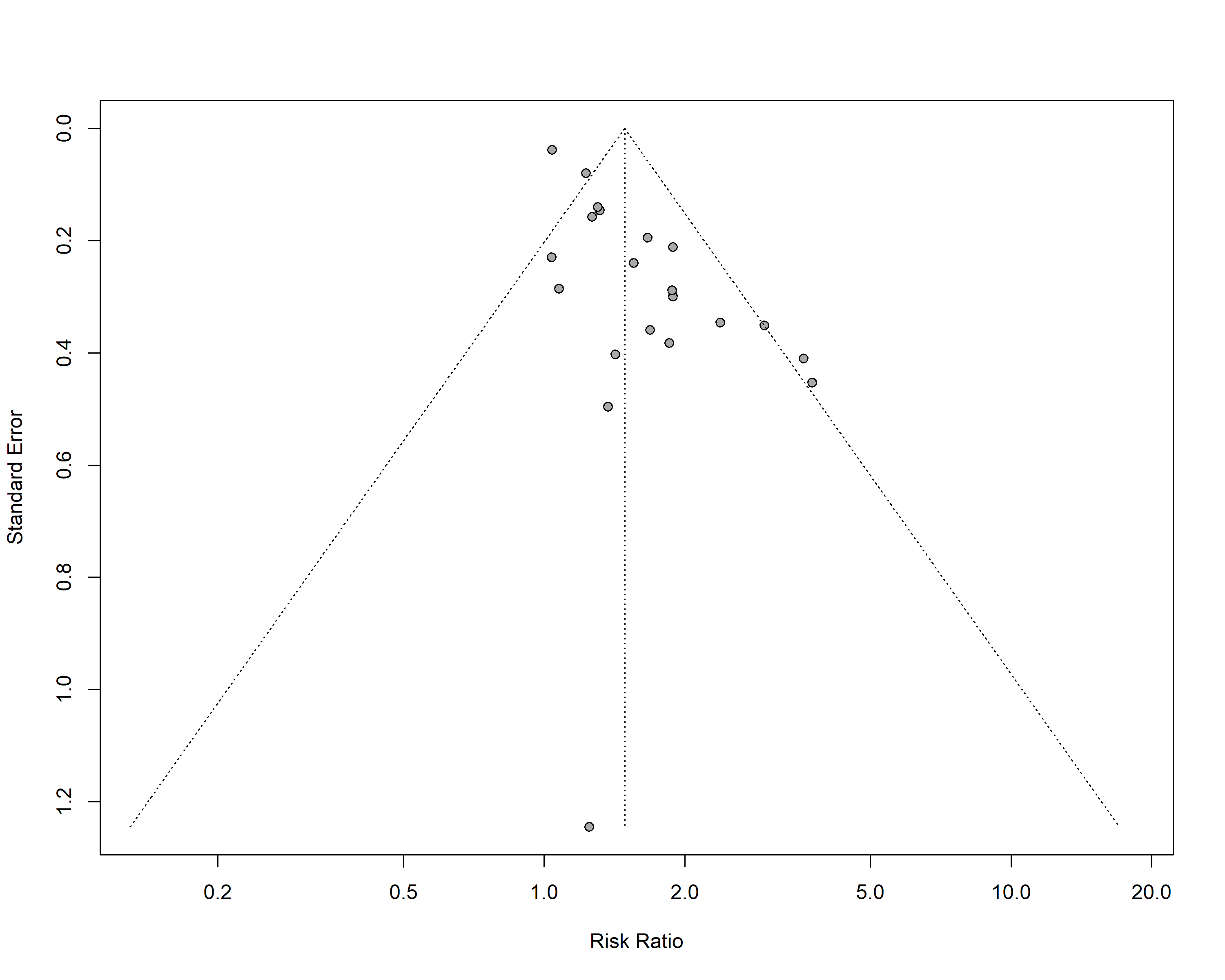
**

**Survival rate**

**
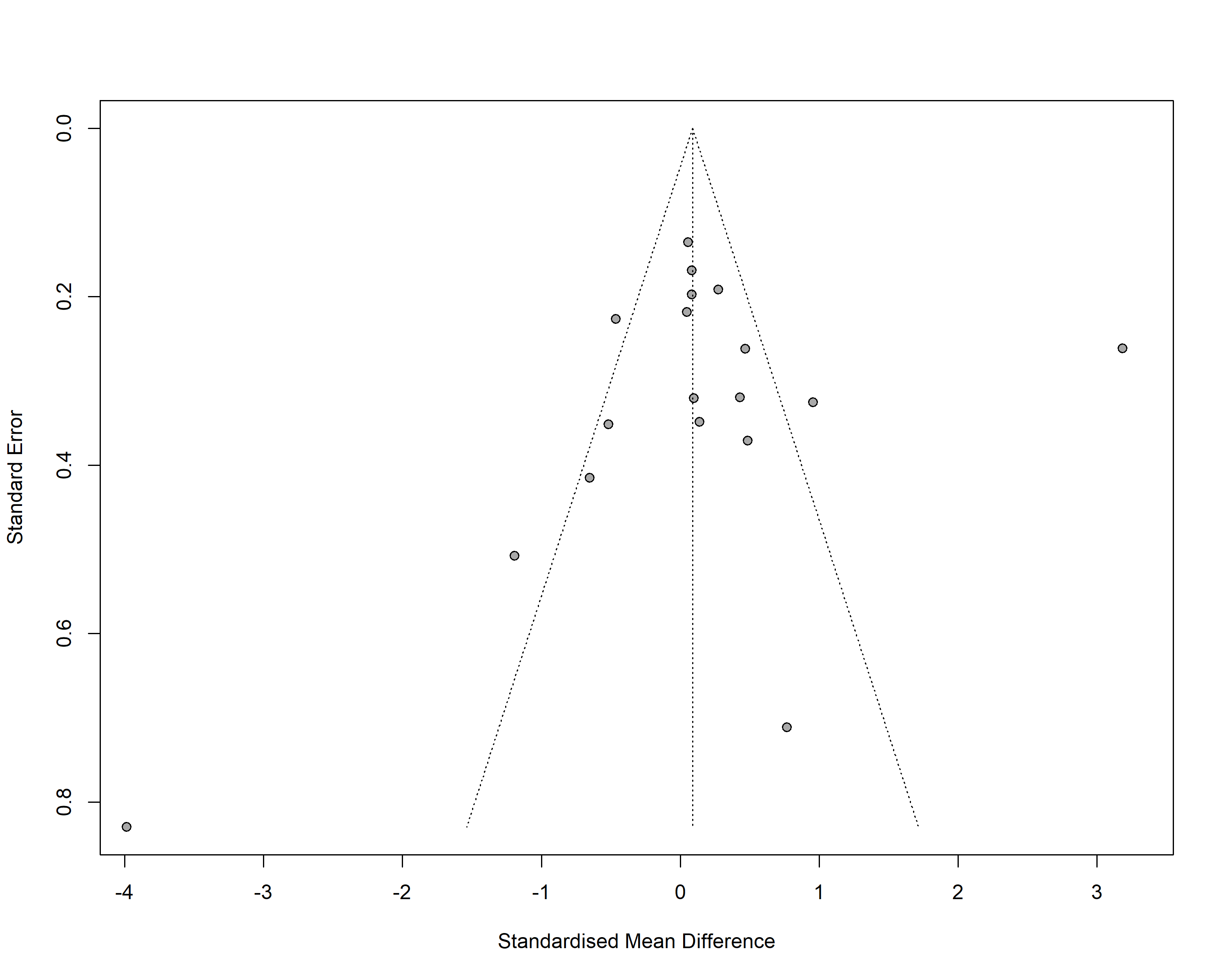
**

**Albumin-RCTs**

**
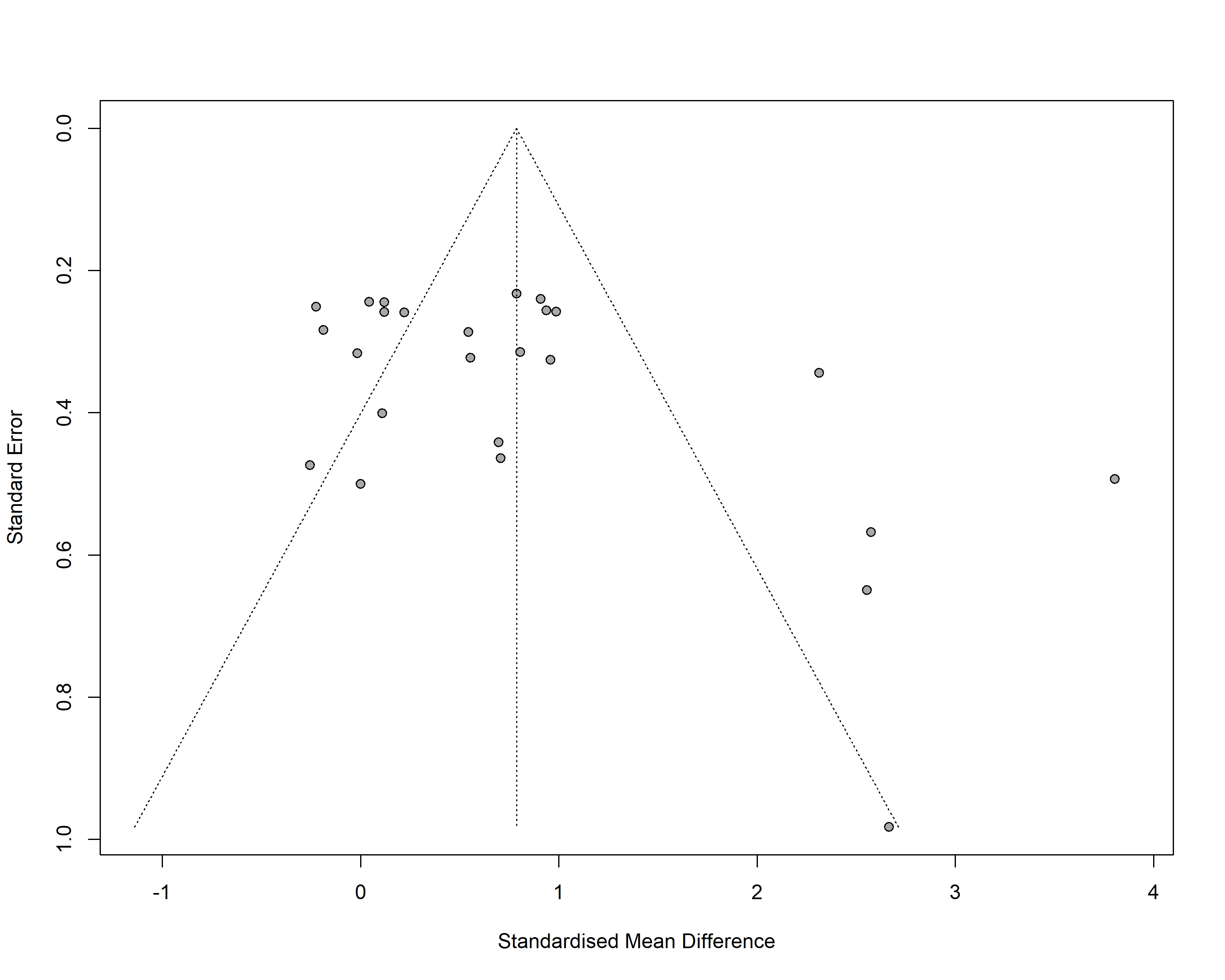
**

**Albumin Non-randomized studies**

**
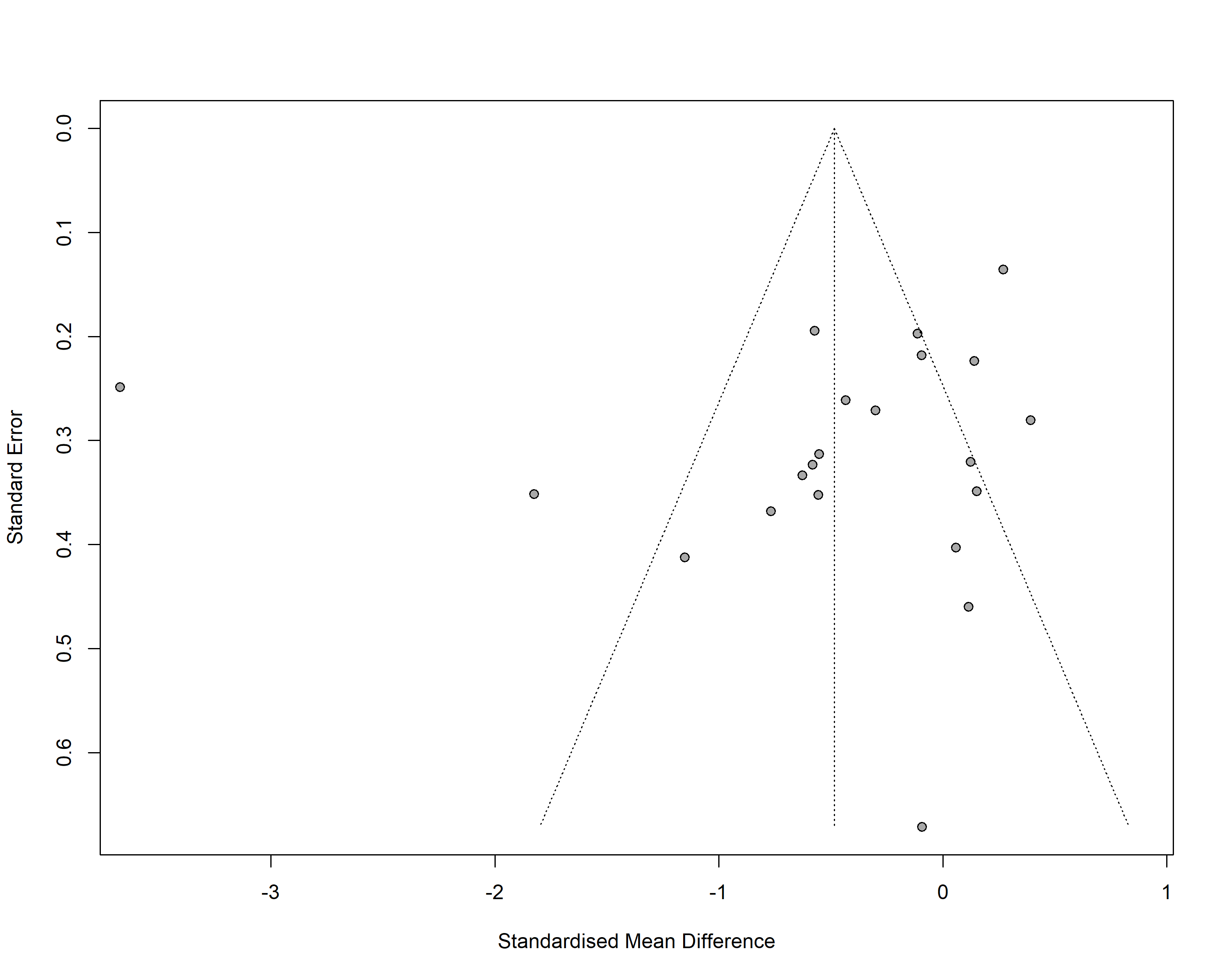
**

**MELD-RCTs**

**
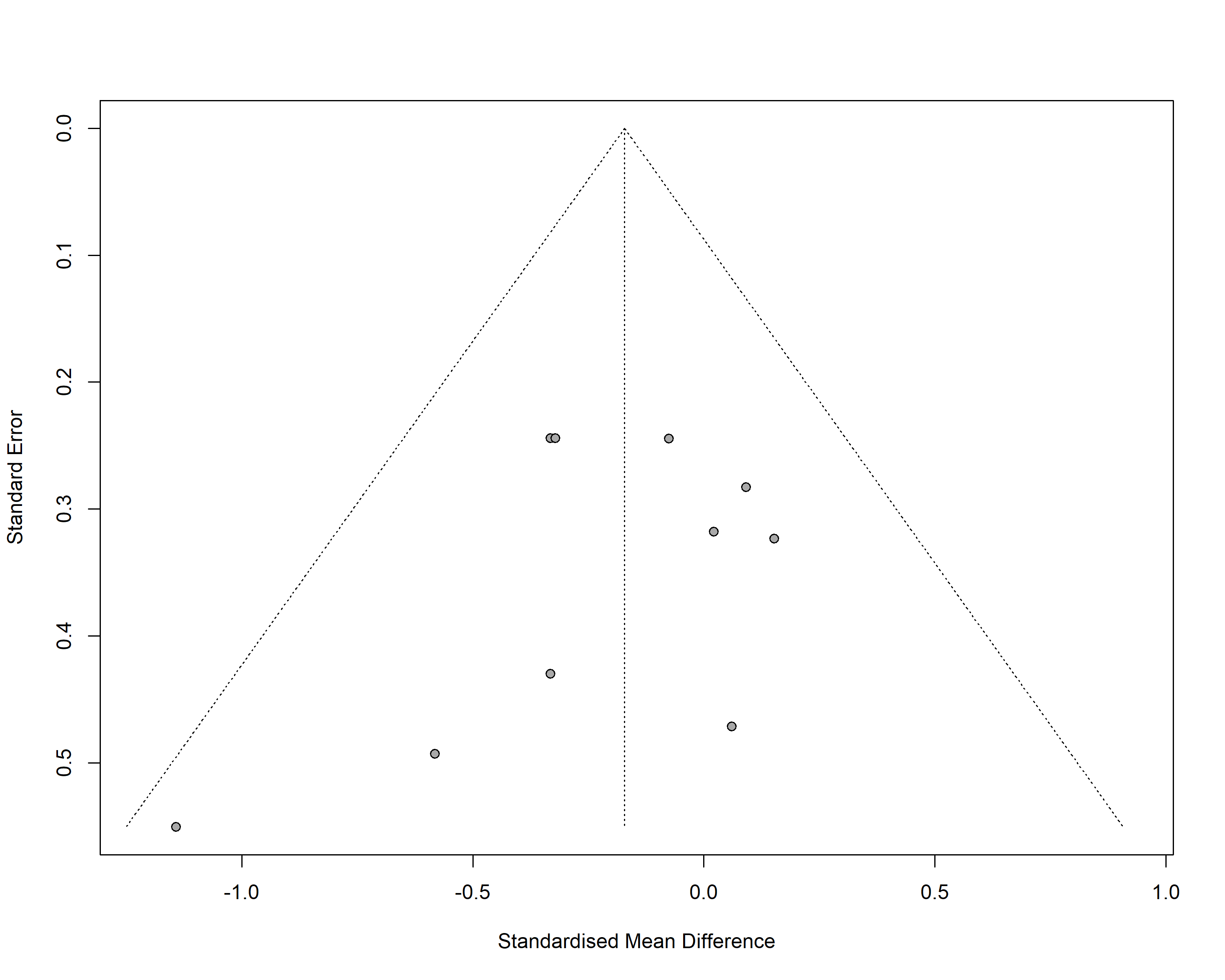
**

**MELD-Non randomized studies**

**
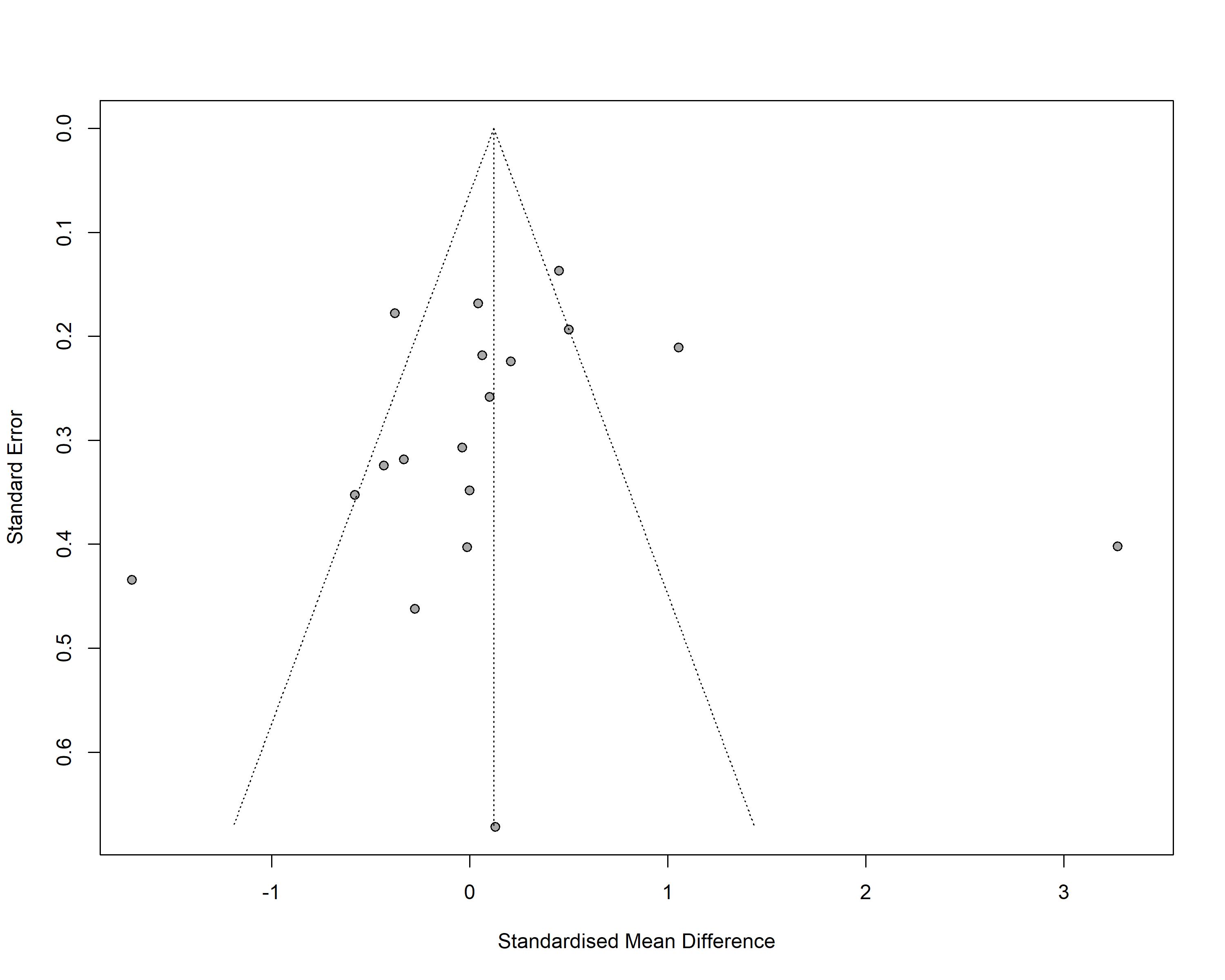
**

**TBIL-RCTs**

**
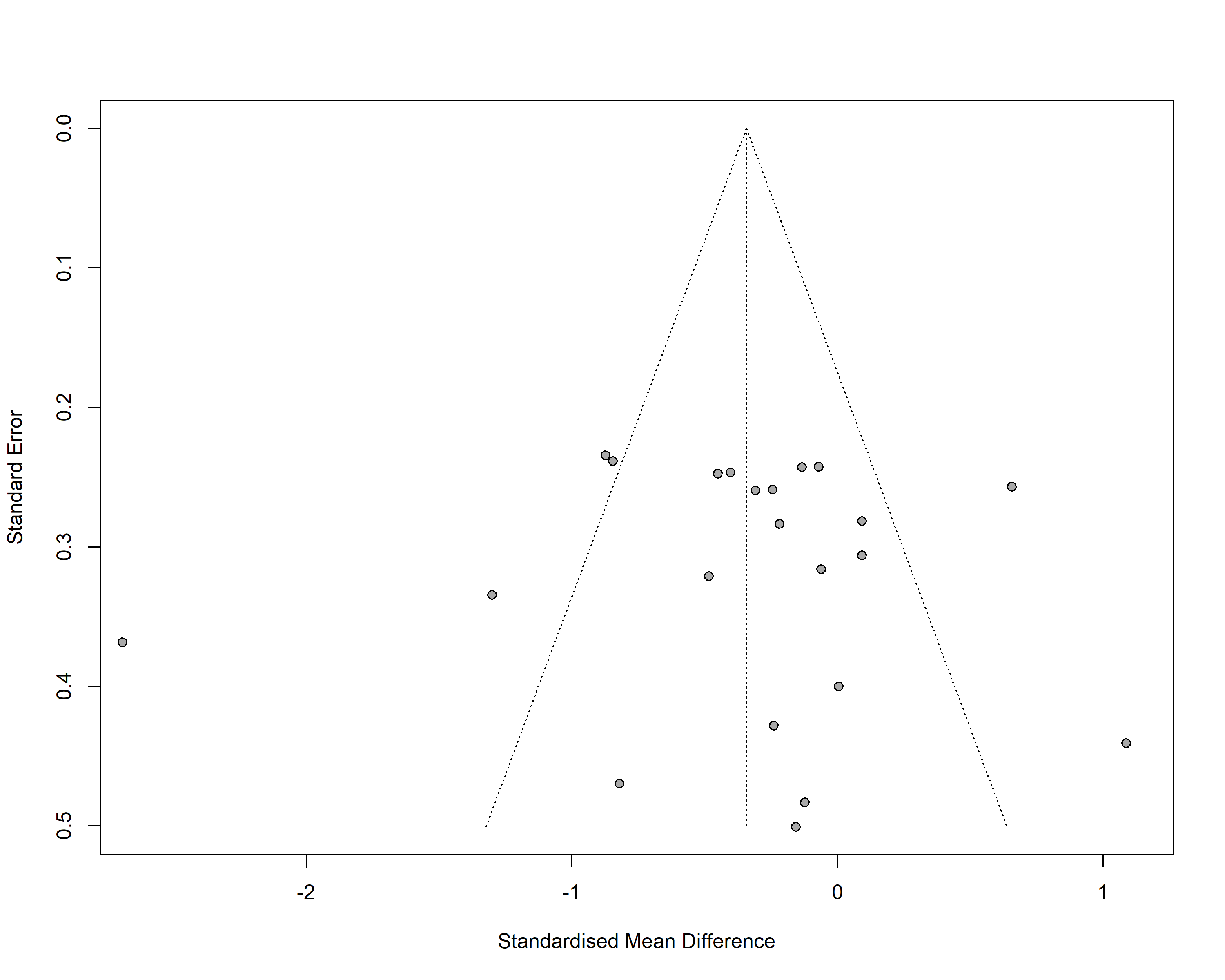
**

**TBIL-Non randomized studies**

**
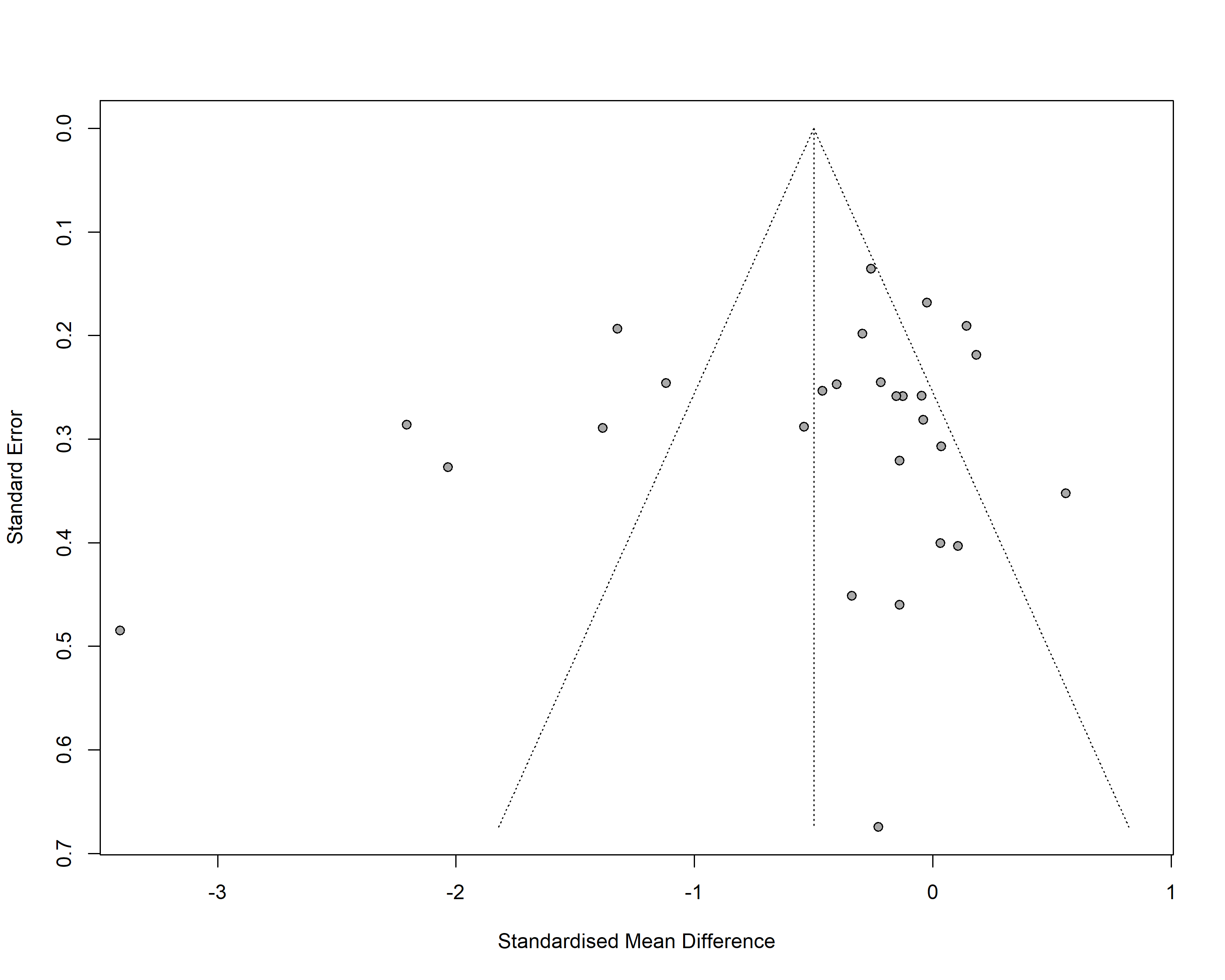
**

**ALT**

**
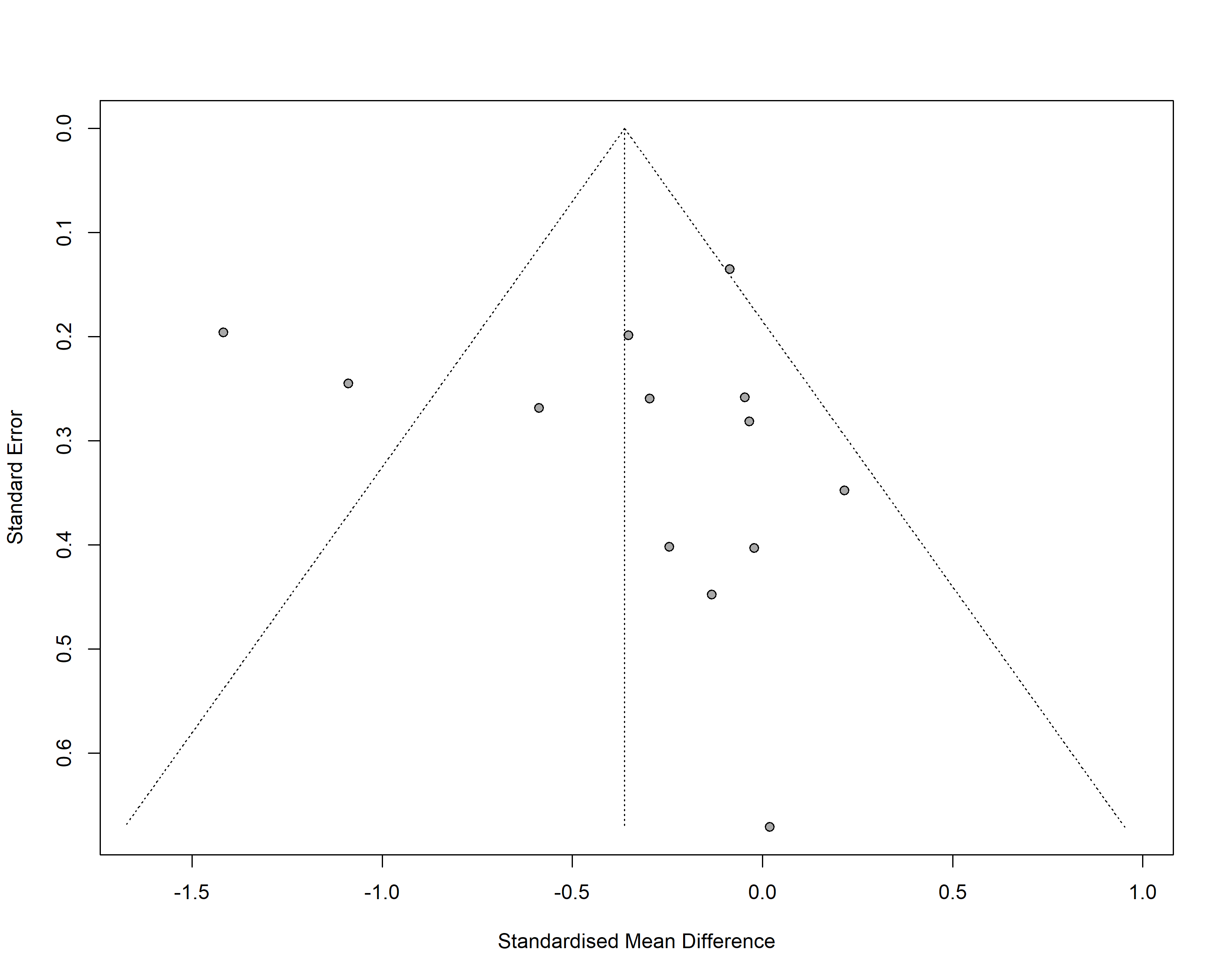
**

**AST**

1. AdiwinataPawitan J. Exploring the most promising stem cell therapy in liver failure: a systematic review. *Stem Cells International*. 2019;2019

2. Chavez-Tapia NC, Mendiola-Pastrana I, Ornelas-Arroyo VJ, et al. Granulocyte-colony stimulating factor for acute-on-chronic liver failure: systematic review and meta-analysis. *Annals of hepatology*. 2015;14(5):631-641.

3. Chen B, Wang Y-H, Qian J-Q, Wu D-B, Chen E-Q, Tang H. Human mesenchymal stem cells for hepatitis B virus-related acute-on-chronic liver failure: a systematic review with meta-analysis. *European Journal of Gastroenterology & Hepatology*. 2018;30(10):1224-1229.

4. Chen B, Pang L, Cao H, et al. Autologous stem cell transplantation for patients with viral hepatitis-induced liver cirrhosis: a systematic review and meta-analysis. *European Journal of Gastroenterology & Hepatology*. 2019;31(10):1283-1291.

5. Huang W, Ma Y, Du L, et al. Effectiveness of granulocyte colony-stimulating factor for patients with acute-on-chronic liver failure: a meta-analysis. *Annals of Saudi Medicine*. 2021;41(6):383-391.

6. Kim G, Eom YW, Baik SK, et al. Therapeutic effects of mesenchymal stem cells for patients with chronic liver diseases: systematic review and meta-analysis. *Journal of Korean Medical Science*. 2015;30(10):1405.

7. Konstantis G, Tsaousi G, Pourzitaki C, et al. Efficacy of Granulocyte Colony-Stimulating Factor in Acute on Chronic Liver Failure: A Systematic Review and Survival Meta-Analysis. *Journal of Clinical Medicine*. 2023;12(20):6541.

8. Liu Y, Dong Y, Wu X, Xu X, Niu J. The assessment of mesenchymal stem cells therapy in acute on chronic liver failure and chronic liver disease: a systematic review and meta-analysis of randomized controlled clinical trials. *Stem cell research & therapy*. 2022;13(1):204.

9. Liu Z, Li J, Li P, et al. Stem cell transplantation for the treatment of liver diseases: A systematic review and meta-analysis. *Turk J Gastroenterol*. 2016;27:499-508.

10. Lu W, Qu J, Yan L, et al. Efficacy and safety of mesenchymal stem cell therapy in liver cirrhosis: a systematic review and meta-analysis. *Stem Cell Research & Therapy*. 2023;14(1):301.

11. Ma X-R, Tang Y-L, Xuan M, Chang Z, Wang X-Y, Liang X-H. Transplantation of autologous mesenchymal stem cells for end-stage liver cirrhosis: a meta-analysis based on seven controlled trials. *Gastroenterology Research and Practice*. 2015;2015

12. Ouyang S, Ouyang L, Li Y, Ye Y, Ban L. Bone marrow-derived stem cells for patients with liver cirrhosis: A systematic review and meta-analysis. *The Turkish Journal of Gastroenterology*. 2021;32(10):896.

13. Pan X-N, Zheng L-Q, Lai X-H. Bone marrow-derived mesenchymal stem cell therapy for decompensated liver cirrhosis: a meta-analysis. *World Journal of Gastroenterology: WJG*. 2014;20(38):14051.

14. Pankaj P, Zhang Q, Bai X-L, Liang T-B. Autologous bone marrow transplantation in decompensated liver: Systematic review and meta-analysis. *World Journal of Gastroenterology: WJG*. 2015;21(28):8697.

15. Qiu B, Liang JX, Romero Gómez M. The efficacy and safety of granulocyte colony-stimulating factor in the treatment of acute-on-chronic liver failure: A systematic review and meta-analysis. *Plos one*. 2023;18(11):e0294818.

16. Rajpurohit S, Musunuri B, Basthi Mohan P, Bhat G, Shetty S. Role of granulocyte colony stimulating factor in the treatment of cirrhosis of liver: a systematic review. *Journal of International Medical Research*. 2023;51(11):03000605231207064.

17. Sang W, Lv B, Li K, Lu Y. Therapeutic efficacy and safety of umbilical cord mesenchymal stem cell transplantation for liver cirrhosis in Chinese population: a meta-analysis. *Clinics and Research in Hepatology and Gastroenterology*. 2018;42(3):193-204.

18. Shi P, Zhang J, Wu M, et al. The effects of granulocyte-colony stimulating factor on chronic liver disease: a meta-analysis. *The Journal of Infection in Developing Countries*. 2022;16(03):537-546.

19. Sun A, Gao W, Xiao T. Autologous bone marrow stem cell transplantation via the hepatic artery for the treatment of hepatitis B virus-related cirrhosis: a PRISMA-compliant meta-analysis based on the Chinese population. *Stem Cell Research & Therapy*. 2020;11:1-17.

20. Tao H, Li Y, Wang T, Zhou C. Umbilical cord blood stem cells transplantation as an adjunctive treatment strategy for liver cirrhosis in Chinese population: a meta-analysis of effectiveness and safety. *Therapeutics and Clinical Risk Management*. 2018:417-440.

21. Wang H, Yao W, Wang Y, et al. Meta-analysis on last ten years of clinical injection of bone marrow-derived and umbilical cord MSC to reverse cirrhosis or rescue patients with acute-on-chronic liver failure. *Stem Cell Research & Therapy*. 2023;14(1):267.

22. Wu C-X, Wang D, Cai Y, Luo A-R, Sun H. Effect of autologous bone marrow stem cell therapy in patients with liver cirrhosis: a meta-analysis. *Journal of Clinical and Translational Hepatology*. 2019;7(3):238.

23. Xue R, Meng Q, Li J, et al. The assessment of multipotent cell transplantation in acute-on-chronic liver failure: a systematic review and meta-analysis. *Translational Research*. 2018;200:65-80.

24. Xue R, Meng Q, Dong J, et al. Clinical performance of stem cell therapy in patients with acute-on-chronic liver failure: a systematic review and meta-analysis. *Journal of Translational Medicine*. 2018;16:1-13.

25. Yang Q, Yang Y, Shi Y, Lv F, He J, Chen Z. Effects of granulocyte colony-stimulating factor on patients with liver failure: a meta-analysis. *Journal of Clinical and Translational Hepatology*. 2016;4(2):90.

26. Zhao L, Chen S, Shi X, Cao H, Li L. A pooled analysis of mesenchymal stem cell-based therapy for liver disease. *Stem Cell Research & Therapy*. 2018;9:1-13.

27. Zhou G-P, Jiang Y-Z, Sun L-Y, Zhu Z-J. Therapeutic effect and safety of stem cell therapy for chronic liver disease: a systematic review and meta-analysis of randomized controlled trials. *Stem Cell Research & Therapy*. 2020;11:1-19.

28. Zhu C-H, Zhang D-H, Zhu C-W, et al. Adult stem cell transplantation combined with conventional therapy for the treatment of end-stage liver disease: a systematic review and meta-analysis. *Stem Cell Research & Therapy*. 2021;12:1-18.
